# Essential statistical principles of clinical trials of pain treatments

**DOI:** 10.1097/PR9.0000000000000863

**Published:** 2020-12-18

**Authors:** Robert H. Dworkin, Scott R. Evans, Omar Mbowe, Michael P. McDermott

**Affiliations:** a Departments of Anesthesiology and Perioperative Medicine, Neurology, and Psychiatry, and Center for Health + Technology, University of Rochester School of Medicine and Dentistry, Rochester, NY, USA; b Department of Biostatistics and Bioinformatics and the Biostatistics Center, George, Washington University, Washington DC, USA; c Department of Biostatistics and Computational Biology, University of Rochester School of Medicine and Dentistry, Rochester, NY, USA; d Departments of Biostatistics and Computational Biology and Neurology, and Center for Health + Technology, University of Rochester School of Medicine and Dentistry, Rochester, NY, USA

**Keywords:** Clinical trials, Statistical analysis, Research design, Endpoints, Outcomes, Missing data, Chronic pain, Acute pain

## Abstract

This article presents an overview of fundamental statistical principles of clinical trials of pain treatments. Statistical considerations relevant to phase 2 proof of concept and phase 3 confirmatory randomized trials investigating efficacy and safety are discussed, including (1) research design; (2) endpoints and analyses; (3) sample size determination and statistical power; (4) missing data and trial estimands; (5) data monitoring and interim analyses; and (6) interpretation of results. Although clinical trials of pharmacologic treatments are emphasized, the key issues raised by these trials are also directly applicable to clinical trials of other types of treatments, including biologics, devices, nonpharmacologic therapies (eg, physical therapy and cognitive-behavior therapy), and complementary and integrative health interventions.

## Abstract 2

Fundamental statistical considerations relevant to phase 2 proof of concept and phase 3 confirmatory randomized trials investigating the efficacy and safety of pain treatments are reviewed.

## 1. Introduction

This article presents an overview for clinician investigators of fundamental statistical principles of randomized clinical trials (RCTs). Our primary objective is to help nonstatisticians understand essential statistical concepts relevant to the design, analysis, and interpretation of clinical trials of pain treatments so that they can collaborate more effectively with their biostatistician colleagues. This article may also be of interest to clinicians seeking to improve their ability to understand and interpret published clinical trials. It is important to emphasize that the information we provide does not substitute for the need to collaborate with biostatisticians when conducting a clinical trial. Indeed, effective collaboration with biostatisticians addresses ethical requirements for data integrity and for clinical trial results to be as informative as possible.^2,4,101^

We discuss 6 sets of issues that we believe are critically important for investigators conducting clinical trials and for others seeking to translate clinical trial results to clinical practice: (1) research design; (2) endpoints and analyses; (3) sample size determination and statistical power; (4) missing data and trial estimands; (5) data monitoring and interim analyses; and (6) interpretation of results. For readers who would like more detailed information about these issues and about other statistical considerations, there are multiple textbooks available.^31,57,66,133,163,170,179^ In addition, documents from the European Medicines Agency and the US Food and Drug Administration (FDA) present regulatory perspectives on evaluating the efficacy and safety of treatments for acute and chronic pain.^51,203^

We focus on phase 2 proof of concept and phase 3 confirmatory randomized trials investigating the efficacy and safety of pharmacologic treatments for chronic pain (we use Arabic numerals when referring to clinical trial phases, as does the FDA; although Roman numerals are also used, the characteristics of these trial phases are the same irrespective of how they are denoted). It is important to emphasize, however, that the key issues raised by clinical trials of medications are also directly applicable to clinical trials of other types of treatments, including biologics, devices, nonpharmacologic therapies (eg, physical therapy and cognitive-behavior therapy), and complementary and integrative health interventions (eg, acupuncture and meditation). In addition, although much of the following material is relevant to prevention and disease modification clinical trials, these types of studies also have specific methodologic and statistical considerations that are beyond the scope of this article.^75,134^

We devote relatively limited attention to the analysis and interpretation of adverse events and to benefit–risk assessments, both of which involve challenging issues and require considerable biostatistical and clinical expertise. Although most treatments for acute and chronic pain are generally safe, almost all have relatively small risks of serious adverse events or poor tolerability in substantial percentages of patients. The difficulty of studying adverse events, unless they are relatively common, is well known. In addition, the relatively brief treatment exposures in almost all RCTs of analgesic medications—typically, several days to a few weeks for acute pain and almost never more than 3 months for chronic pain—further limit the conclusions that can be drawn about safety. In the community, patients with chronic pain can be taking analgesic medications on a daily or intermittent basis for years, possibly increasing the risk of rare but serious adverse outcomes if such events are associated with the treatment.

Before proceeding further, the importance of registering clinical trials on authoritative websites such as www.clinicaltrials.gov must be emphasized. Prospective registration of clinical trials before the beginning of enrollment—with updating of information if protocols or prespecified analysis plans are revised and when results become available—has multiple benefits. Perhaps most importantly from a statistical perspective, these include reducing selective reporting of analyses and outcomes and preventing publication bias from failure to report the results of clinical trial.

## 2. Research design

### 2.1. Identifying the objectives of the clinical trial

The first step in designing a clinical trial is to identify the objectives of the trial. In considering the approval of medications, the FDA emphasizes that the beneficial effects of a medication involve how patients feel, function, or survive. Many RCTs examine the effects of a treatment on at least 1 of these 3 types of outcomes. Because clinical trials involve a substantial amount of effort and can require appreciable financial and other resources, most are designed to obtain data relevant to several objectives, for example, to evaluate the effect of a treatment on pain and on physical function as well as to determine its safety and tolerability. When there are multiple objectives, they should be prioritized and accompanied by statistical formulations of the questions. This often involves prespecified hypothesis testing, although some studies, particularly early-phase trials, may not involve tests of specific hypotheses.

Clinical trials are often categorized as phase 1, 2, 3, or 4 studies. Various guidelines and definitions exist for these trial phases (eg, www.fda.gov/ForPatients/Approvals/Drugs/ucm405622.htm), but the boundaries between them are not rigid and available definitions are not completely consistent; indeed, the characteristics and application of these phases can be somewhat different depending on whether the terminology is being used by regulatory agencies, government or foundation funding organizations, or academic investigators. In general, phase 1 trials are “first-in-human” studies that are designed to provide an initial evaluation of safety and drug pharmacokinetics, often conducted in healthy volunteers. Phase 2 trials are typically the first studies conducted in patients with the specific condition for which the treatment is intended, and they provide further information regarding safety, target engagement, route of administration, and dosage, as well as preliminary evidence of efficacy. Studies that seek preliminary evidence of efficacy are sometimes referred to as “proof of concept” clinical trials, and most phase 2 trials of analgesics for acute or chronic pain examine multiple dosages and have total sample sizes of between 50 and no more than 300 patients.

Phase 3 trials, also referred to as “confirmatory trials,” are designed to determine whether there is convincing evidence of efficacy; such trials include the types of studies required for regulatory approval by, for example, the FDA or European Medicines Agency. These trials also provide information addressing longer-term safety and tolerability in larger samples, typically 300 to 800 patients for acute and chronic pain conditions. In certain circumstances, phase 3 trials of pain treatments could have sample sizes substantially larger than this. For example, a trial designed to evaluate cardiovascular risks associated with a novel nonsteroidal anti-inflammatory drug might require thousands of patients.^147^

Phase 4 trials are usually conducted after a treatment is available to patients in the community. They are often intended to provide additional evidence of efficacy or safety, for example, using different outcome measures, examining specific subgroups of patients such as the elderly, evaluating longer treatment durations, or assessing cost-effectiveness. Randomized clinical trials conducted in different conditions than those for which regulatory approval has been granted are sometimes referred to as phase 4 trials; for example, a study of an analgesic medication in patients with pain associated with multiple sclerosis after it has been approved for painful diabetic peripheral neuropathy. However, depending on their objectives, such studies could also be considered phase 2 or 3 trials.

In discussing these clinical trial phases, we focus on the extent to which efficacy and safety are examined. The efficacy of an investigational treatment is typically evaluated by examining whether it provides statistically significantly greater benefit when compared with a comparison intervention. For RCTs of analgesic medications, the comparison intervention is usually matching placebo (typically inert, but sometimes a medication that mimics the side effects of the active treatment to mitigate unblinding from side effects or their absence). For studies of devices and other invasive treatments, the comparator could be a “sham” device or intervention. Comparison interventions can also be another active treatment. For example, efficacy can be demonstrated by showing that an investigational treatment is associated with greater benefit than an existing treatment with well-established efficacy.

The term “effectiveness” is also widely used, but we will use it only when referring to studies that attempt to evaluate how beneficial the treatment would be when administered in clinical practice. Such trials include samples of patients who are more heterogeneous than those typically examined in phase 2 and 3 trials and are more likely to allow patients to initiate or continue other treatments for their condition. For example, a trial could be designed to examine the effectiveness of an analgesic medication in a large sample of patients with chronic low back pain that includes those who have other pain conditions and psychiatric comorbidities, receive workers' compensation benefits, or are involved in litigation, all of which are very often exclusion criteria in phase 2 and 3 trials examining the efficacy of chronic pain treatments. The results of effectiveness trials often have greater generalizability (ie, external validity) than the results of trials evaluating efficacy, which typically seek to reduce variability and bias to the greatest extent possible.

Our discussion of clinical trials to this point has assumed that evaluations of efficacy will be tests of the scientific hypothesis that one treatment is associated with a greater reduction in pain than another, which is generally referred to as a test of “superiority.” It is important to note that what is actually being tested, when formulated as a statistical hypothesis, is a null hypothesis of no difference between the 2 treatments in, say, their mean pain intensity at the end of the trial. When, on the basis of the observed data, the null hypothesis is rejected at a prespecified significance level α, it can be concluded that the data provide support for the alternative hypothesis of a difference in mean pain intensity at the end of the trial between the 2 treatments.

When treatments with very well-established efficacy exist, a noninferiority trial^64,204^—or less commonly, an equivalence trial^103^—may be conducted. A noninferiority trial design can be used to show that a new treatment is not worse by more than a prespecified amount than an established treatment for a specific endpoint and is designed to test the null hypothesis that the difference between the 2 treatments (established − new) is greater than a prespecified noninferiority margin, which is typically selected on the basis of clinical considerations and historical data. Rejection of the null hypothesis on the basis of the observed data implies that the data provide support for the alternative hypothesis that the difference between the 2 treatments (established − new) is no greater than the noninferiority margin. Noninferiority designs can negate the need for placebo groups, which cannot be used when evaluating treatments for life-threatening or rapidly progressive conditions for which efficacious interventions already exist. By contrast, an equivalence trial is used to determine whether a new treatment is “no better and no worse” than an established treatment; such trials are commonly used to establish bioequivalence between, for example, a brand-name product and a generic version with respect to pharmacokinetic parameters.^178,179^

Because any shortcomings in the design, execution, and analysis of equivalence and noninferiority trials will tend to bias the results toward showing equivalence or noninferiority, these types of trials must be as methodologically rigorous as possible.^64,204^ In addition, there is a major issue associated with these clinical trial designs that may limit their use for studying analgesic medications and other types of pain treatments. Because a noninferiority or equivalence clinical trial typically does not include a placebo group, the conclusion of noninferiority relies on an important assumption, namely that both the new treatment and the established treatment would have been shown to be superior to placebo had a placebo group been included. This assumption would be supported by consistent demonstration of the superiority of the established treatment to placebo in multiple trials. However, some conditions, such as pain and many psychiatric disorders, are prone to variable and sometimes prominent placebo effects, making the results of noninferiority trials difficult to interpret. It could be, for example, that neither treatment would have been shown to be efficacious if a placebo group had been included in the trial.^122^ Indeed, although there are many analgesic medications with well-established efficacy, few if any demonstrate efficacy consistently.^40,43,62^

One solution to this problem is to include a placebo group in these trials to establish “assay sensitivity,” that is, the ability of a clinical trial “to distinguish an effective treatment from a less effective or ineffective treatment.”^50^ If efficacy of the established treatment vs placebo is shown, it can be assumed that the trial has adequate assay sensitivity to conduct an informative test of the noninferiority or equivalence of the investigational treatment vs the established treatment.^14^

### 2.2. Addressing major sources of bias

#### 2.2.1. Randomization

Randomization makes it possible to draw causal inferences on the basis of the results of an RCT and to conclude that outcome differences between, for example, an active treatment and a placebo group have been caused by the active treatment. The major goal of randomization is to create groups of patients who are as similar as possible except for the intervention assignment. In a parallel group RCT with a sufficiently large sample of patients, randomization is expected to result in similar distributions of patient characteristics among the groups, including those measured and unmeasured, as well as known and unknown. Randomization of smaller groups of patients is less likely to result in comparability of the groups, which could potentially explain group differences in the outcome of treatment. For example, if patients in an active treatment group have, on average, a milder condition with a better prognosis than those in the placebo group, differences in outcomes that are explained by patient characteristics could be erroneously attributed to the treatment.

Randomizing patients in a clinical trial also eliminates both intentional and unintentional bias in the allocation of treatments to patients, which could compromise the validity of clinical trial results by, for example, allocating patients expected to improve to the investigational treatment rather than placebo. Indeed, in meta-regression analyses of 234 meta-analyses of almost 2,000 trials, treatment effect estimates appeared to be exaggerated in RCTs with inadequate or unclear random-sequence generation, and this effect occurred primarily in trials with subjective outcomes.^175^ Bias in the allocation of patients to treatments is eliminated by prespecifying a randomization protocol and by preventing study staff from having any information about the specific treatment group to which patients in a trial will be assigned. Allocation concealment is intended to prevent selection bias—that is, the assignment of certain patients to specific treatments—and can be used even in trials in which other aspects of blinding might be difficult to implement, such as a trial comparing pharmacologic and nonpharmacologic treatments.^177^

Two additional aspects of randomization that are commonly used are blocking and stratification. Blocking is a method for limiting imbalances in the number of participants assigned to each group after a certain number of participants have been enrolled. For example, in a study with treatment groups A and B, the first 4 patients could be randomized in any potential combination that would produce an equal number of patients in each of the 2 groups (eg, ABAB and BBAA). After the first block is complete, the next 4 patients would be assigned to the 2 treatments using a newly randomized sequence of length 4 (block size) and so on. Although in this example the block size is 4, it can be any number that is a multiple of the number of treatment groups. Use of a small block size is advantageous in terms of promoting equal allocation over short periods of time, but it may make it easier for trial staff to correctly guess the treatment assignments. One can use block sizes that vary randomly (eg, a combination of blocks of 4 and 6 treatment assignments). If blocking is incorporated in the randomization plan, to avoid any compromise of the blind, it is important to not reveal the block size(s) in the protocol.

Stratified allocation can be used to promote comparability of the treatment groups with respect to factors that are known to be associated with outcome. For example, if depression is thought to be associated with outcome, patients can be separated into those who are and are not depressed and randomized within each stratum. In multicenter trials, center is commonly chosen to be a stratification factor to prevent the chance occurrence of most of the participants at that center being randomized to one of the treatment groups. It is generally recommended that the number of stratification variables be small and limited only to those known to have important associations with outcome. In particular, it is important to avoid small strata because chance imbalances in treatment group allocation within a stratum are more likely in that case, and stratification can be self-defeating if such imbalances accumulate across strata.^165^ Combining stratification with blocking, that is, using blocking within strata, can be especially helpful in this case.

#### 2.2.2. Blinding

Blinding in clinical trials refers to when one or more parties, including patients, clinicians, or research staff, are unaware of the treatment arm to which study subjects are assigned. In a single-blind trial, either the patients or the clinicians/research staff are unaware of treatment assignment, whereas in a double-blind trial, both the patients and the clinicians/research staff are unaware of treatment assignment. It is well established that expectations can have a powerful effect on human behavior and be a major source of bias in clinical trials; this may be especially true in clinical trials of pain treatments given the prominent role of subjective outcome measures. In one meta-analysis, treatment effect estimates were on average 13% greater in trials in which there was no or inadequate blinding compared with double-blind trials.^175^ The bias associated with a lack of adequate blinding was greater with subjective outcomes such as pain, mood, and quality of life than it was with objective outcomes and mortality.^175^ The results of double-blind RCTs are typically less subject to bias and more informative than the results of unblinded studies.

The results of a single-blind trial are more subject to bias than those of a double-blind trial because unblinded research staff could unintentionally or intentionally communicate their expectations to patients or influence treatment outcomes in other ways, thereby making a treatment appear to be effective when it is not. For some treatments, such as physical therapy, or invasive interventions such as surgery, blinding of the research team and patients may be impossible. In many of these situations, however, the individuals conducting outcome assessments can be kept blind to the patient's treatment, which can limit some but not all sources of bias. Treatment effect estimates are generally greater in clinical trials with inadequate investigator and patient blinding,^97,148,213^ and the results of unblinded or “open-label” trials can overestimate effectiveness and potentially be misleading.

When it is not possible to conduct a clinical trial on a fully double-blind basis, efforts should be made to ensure that patient and investigator expectations are as neutral as possible.^35^ For example, there should be no communications or materials—including the informed consent form—that suggest that one treatment is newer, is better, or has fewer side effects than the other. Although relatively little attention has been paid to managing patient and research staff expectations in clinical trials of pain treatments, there is growing recognition of the importance of patient and research staff training and ongoing monitoring as a means of increasing the quality of clinical trial data.^184,196^

Typically, the placebo in studies of analgesic medications is inert but appears identical to the active medication in color, shape, size, taste, and even odor. This is a critical aspect of keeping patients and research staff blinded with respect to the treatment that the patient is receiving. Even in double-blind RCTs, however, patients and investigators can sometimes accurately guess which intervention patients are receiving, either because of characteristic side effects or because the treatment seems to be beneficial. Following completion of participation, patients and investigators can be asked which intervention they believe was received (or, in the case of cross-over trials, what the treatment sequence was) and what is the basis of their guesses.^145^ In a clinical trial of an efficacious treatment, patients could correctly guess that they received the active treatment because of its beneficial effects, which would not be evidence of compromised blinding. It is only when patients correctly guess their treatment based on factors that are unrelated to efficacy, such as side effects, that the adequacy of the blinding and the potential of bias must be considered.

Some RCTs of analgesic medications have used “active placebos,” which are medications that have no known pain-relieving effects but that have side effects that mimic those of the analgesic medication being studied (eg, sedation and constipation). The use of active placebos can be an effective strategy for maintaining patient and investigator blinding, perhaps especially in cross-over trials in which patients, being exposed to all study interventions, may be more likely to correctly guess when they received an inert placebo and when they received an active treatment. The use of active placebos, however, remains somewhat controversial because of the ethical issues involved in exposing patients to the risk of side effects but not to the potential benefit of receiving the active medication. It has been argued that in studies of antidepressant medications in depressed patients, “the available evidence does not provide a compelling case for the necessity of an active placebo.”^169^ Given the difficulty of identifying what could serve as an active placebo for many of the medications studied for the treatment of pain, it would be valuable to attempt to determine whether active placebos increase the validity of analgesic clinical trials or whether they are unnecessary.

### 2.3. Major types of clinical trials

Several different types of clinical trial designs have been used for testing whether a treatment hypothesized to relieve pain has superior benefits compared with a control intervention. Most of these designs can also be used to examine group differences in adverse events and safety risks. The designs discussed in this section can also be used to test noninferiority or equivalence, although there have been few such RCTs of pain treatments.

#### 2.3.1. Parallel group designs

The most common type of clinical trial of pain treatments is the parallel group design, in which patients are randomized to 2 or more treatments, one of which is usually placebo or another comparator that is expected to have no or minimal pain-relieving properties. Causal inference from the results of a double-blind parallel group RCT can be quite straightforward. If data integrity and trial quality can be assumed, a statistically significant difference in the primary outcome measure between the active intervention and the control condition can be interpreted as evidence that the treatment caused the difference.

#### 2.3.2. Cross-over designs

In cross-over trials, each patient is randomized to 1 of 2 or more treatment sequences. For this reason, cross-over clinical trials should only be considered when the condition being treated is expected to remain stable throughout the duration of the trial, the treatment being investigated has a relatively prompt onset of action, and the effect of the treatment disappears relatively soon after treatment withdrawal. In most cross-over trials, there is a “washout period” between the different treatment periods to allow any effects of the earlier treatment to dissipate before starting the next treatment. In a typical analysis of the data from a 2-period placebo-controlled cross-over trial, patient outcomes at the end of their active treatment period are compared with their outcomes at the end of the placebo period.

Compared with parallel group trials, cross-over trials can be very efficient with respect to sample size requirements because each patient receives both active and control treatments, which removes the between-patient variability that is present in parallel group trials. Unfortunately, cross-over trials also have several potential limitations. The cross-over design assumes that differences in outcome between the treatments do not depend on the period in which the treatments are given, that is, there is no interaction between the treatment and period. If the pain condition being studied changes over the course of the trial (eg, pain severity increases), the outcomes in later treatment periods will differ from those in earlier periods, that is, there will be a “period effect.” This may induce an interaction between treatment and period if the magnitude of the treatment effect depends on the severity of the condition. Another potential cause of such an interaction is inadequacy of the length of the washout period whereby the effect of the treatment in the first period, for example, may carry over to the placebo condition in the next period, thus reducing the estimated magnitude of the treatment–placebo difference in the second period. Although various approaches to addressing the presence of these so-called carry-over effects have been proposed, their value remains controversial.^104,178^ In addition, if the treatment is disease-modifying (eg, associated with persisting reduction of pain), then treatment effects in later treatment periods will be attenuated. Despite their limitations, cross-over trials have been very informative designs in evaluating the efficacy of various chronic pain treatments.^42,76^ However, relatively prompt resolution of pain limits the use of cross-over trials for most acute pain conditions.

#### 2.3.3. Enrichment designs

There has been increasing attention to the use of enrichment designs in the study of treatments for chronic pain. One reason for this has been the belief that enrichment can increase the assay sensitivity of a trial to detect efficacy. The most common type of enrichment design in the study of chronic pain has been termed “enriched enrollment randomized withdrawal.”^107,136^ In this design, there is an initial enrichment phase of several weeks in which patients typically receive the investigational treatment on an open-label basis. At the end of this phase, patients whose pain has decreased (eg, by 30% or more) and who have tolerated the treatment are then randomized on a double-blind basis to continued active treatment or to switch to placebo. In this randomized withdrawal phase of the trial, any pharmacologic benefit of an efficacious treatment shown in the open-label phase is expected to continue in the patients who remain on treatment but is expected to dissipate in the patients randomized to placebo. It is hypothesized either that pain will be greater in the placebo vs the active treatment group at the end of the double-blind phase or that the placebo group will have a faster time to a clinically meaningful increase in pain than the active group.

Multiple RCTs of various chronic pain conditions using different classes of medications have used this design, and the methodologic aspects of these trials have been reviewed.^144^ The results of published trials suggest that the assay sensitivity of enriched enrollment randomized trials may be greater than the assay sensitivity of standard parallel group trials, but the evidence is not conclusive.^71,107,144^ However, because the initial open-label phase is typically used to exclude patients with poor tolerability and clinically important adverse events, the subsequent double-blind phase can show a reduced rate of adverse events,^71^ which likely translates to lower rates of withdrawals and missing data. Potential limitations of the enriched enrollment randomized withdrawal design include unblinding due to knowledge of benefits and side effects from the open-label phase as well as the lack of generalizability of the results because only those patients who exhibited a favorable response and tolerability during the open-label phase are randomized.^109,123^ It can also be argued, however, that the design mirrors clinical practice because those who are randomized represent the patients who would continue treatment in practice. Clinical trial designs that include enriched enrollment and randomized withdrawal can play a role in regulatory approval and have been used in phase 2 and 3 trials of chronic pain treatments.^51,144,207^

There are other uses of enrichment in RCTs, for example, for excluding patients who develop dose-limiting adverse events, who have poor medication or pain diary adherence, who have a low risk of outcome events, or who are apparent placebo responders.^114,207^ It is very likely that the use of enrichment in clinical trials of pain treatments will increase as a result of the great interest in developing “precision” or personalized treatments that target specific pathophysiologic mechanisms or biomarkers^69^ rather than broad disease etiologies in which patients appear to have multiple but incompletely shared underlying pain mechanisms.

#### 2.3.4. Factorial designs

Factorial clinical trial designs can be used to simultaneously study the efficacy and safety of 2 or more treatments and examine whether any beneficial (or adverse) effects of the treatments are additive, subadditive, or synergistic. In the most common type of factorial design, patients are randomized to the 4 possible combinations of 2 treatments and their controls. For example, Foster et al.^67^ conducted such a 2 × 2 factorial trial of oral desipramine and topical lidocaine in women with vulvodynia. They randomized patients with equal allocation to receive oral desipramine plus topical lidocaine; oral desipramine plus topical placebo; oral placebo plus topical lidocaine; and oral placebo plus topical placebo. This design makes it possible to test the *main effect* of desipramine on pain by comparing patients randomized to oral desipramine (combined with either topical lidocaine or topical placebo) with patients randomized to oral placebo (combined with either topical lidocaine or topical placebo) and to similarly test the main effect of topical lidocaine. Such comparisons, however, assume that the effect of oral desipramine does not depend on whether or not topical lidocaine is also given, and that the effect of topical lidocaine does not depend on whether or not oral desipramine is also given, that is, there is no interaction between the 2 treatments. The interaction between the 2 treatments can also be tested to determine whether any beneficial effects of the treatments interact synergistically (ie, the benefit of the combination of treatments is greater than what would be expected from the sum of their independent effects, also termed superadditivity) or whether there is subadditivity (ie, the benefit of the combination is less than what would be expected from the sum of their independent effects), which could result from overlap in their mechanisms of action, noncompliance, and other factors.^16^

Factorial designs can be viewed as an efficient way to conduct 2 or more trials for the price of one, but only if it is assumed that there is no interaction between the treatments. Planning such a study should be done with caution because the main effects of treatment can be misleading in the presence of an interaction. In the example above, the effect of oral desipramine vs oral placebo in a standard 2-arm trial would differ from the main effect of oral desipramine in a trial with a 2 × 2 factorial design if an interaction between the 2 treatments was present. Also, although the assumption of no interaction can be tested, the power of the test is low compared with the power of a test for a main effect, so a trial designed to detect main effects may not be able to detect important interactions between the treatments.^16^ If one is to assume the absence of an interaction, there should be sufficient understanding of the treatments to establish confidence that their mechanisms of action are nonoverlapping and that ceiling effects for the improvement with one or both of the treatments are unlikely. In addition, sufficient consideration should be given to potential safety concerns and logistical issues that might promote lower compliance in those assigned to receive combination treatment.

On the other hand, interest may center on interactions between the treatments, in which case comparisons among the individual treatment arms/combinations would be performed. This would require larger sample sizes than a design to detect main effects of a treatment. Given the modest efficacy of all existing treatments for acute or chronic pain as monotherapy, factorial designs can be used to investigate whether combinations of medications or combinations of medications with nonpharmacologic treatments can improve patient outcomes. Complex factorial designs can be used to study multiple interventions^17^; for example, Apfel et al.^5,6^ conducted a 2 × 2 × 2 × 2 × 2 × 2 factorial trial of 6 different interventions for the prevention of postoperative nausea and vomiting.

#### 2.3.5. Adaptive designs

There are multiple types of adaptive clinical trial designs, but their defining characteristic is “prospectively planned modifications to one or more aspects of the design based on accumulating data from subjects in the trial.”^206^ Coffey^25^ and Coffey et al.^26^ distinguish among adaptive designs for early-stage exploratory development, for later-stage exploratory development, and for confirmatory clinical trials. Within these broad categories, the uses of adaptive designs can include (1) identifying a maximum tolerated dose (eg, using the continual reassessment method^72^); (2) selecting a target dosage to study in confirmatory trials (eg, using adaptive dosage allocation); (3) evaluating the assumptions used in sample size calculations (eg, by blinded or unblinded assessments of outcome measure variability or event rates) and modifying the sample size if warranted; (4) interim monitoring to consider early stopping for safety, efficacy, or futility (eg, using group sequential methods); (5) bridging phases 1 and 2 or phases 2 and 3 with adaptive seamless designs (eg, using phase 2 dose-finding data to seamlessly transition to a confirmatory trial); and (6) response adaptive randomization to increase the percentage of patients randomized to one or more treatments showing favorable trends.^9,13,57,206^

Adaptive designs can have advantages and, depending on the specific study hypothesis and trial circumstances (eg, clinical condition and type of treatment), are helpful to consider when beginning to design a clinical trial of a pain treatment. One benefit of certain adaptive designs can be greater efficiency because of smaller overall sample sizes or shorter overall study durations, although such benefits do not always occur.^115,195^ In addition, adaptive designs may increase the likelihood of achieving the trial's objective and provide improved understanding of treatment effects. Adaptive designs may also have particular value in studying rare conditions for which the number of potential research patients in the population is limited, as well as advantages in the investigation of precision or personalized treatments, that is, treatments with greater efficacy or safety in certain subgroups of patients identified on the basis of aspects of their genotype or phenotype.^209^

Because adaptive designs rely on analyses of accumulating data, they sometimes require outcomes that occur relatively early in the course of the treatment, which allows data to be analyzed promptly and any adaptations to then occur as planned. For this reason, disease-modifying treatments with beneficial effects that are expected to occur over several years may not be suitable for an adaptive design, whereas trials of symptomatic treatments of acute or chronic pain could be. Similarly, when temporal changes are expected over the course of the trial in the characteristics of the patients (eg, a new treatment becomes widespread in the community) or of the investigational treatment (eg, surgical technology improves), adaptive designs should not be used because trial modifications will be based on patient and treatment characteristics that could have changed substantially by the time the adaptations are implemented.

There are also many logistical and procedural challenges with implementing adaptive designs. These include issues involving medication (or other intervention) supply and management; data quality, extraction, and analysis; and site and data monitoring.^73^ A critically important aspect of all adaptive designs is the need to prespecify the circumstances in which alterations in the trial will occur and the specific nature of those adaptations. This is needed to ensure that any such trial modifications do not cause unacceptable increases in the type I error probability or compromise trial integrity or data quality.^206^ Within the prespecified description of the characteristics of an adaptive design, it is important to identify whether the adaptations will be based on blinded data (eg, interim sample size re-estimation using the estimated pooled SD) or unblinded data (eg, interim futility analyses). Any examination of unblinded data must be described and potential threats to the integrity of the trial carefully evaluated. Depending on the specific type of adaptive design, there are other potentials for bias. For example, investigators can become unblinded to emerging trends because sample size adjustments based on interim estimates of treatment effects can be reverse engineered to estimate the trend that caused the adjustment.^34^ To mitigate such sources of unblinding, firewalls to prevent investigators from knowing that an adaptation has occurred should be implemented whenever circumstances permit. Because clinical trials with adaptive designs are usually more complex than standard RCTs, biostatisticians with substantial expertise are required for their design, and interpretation of their results can be challenging for readers who are not familiar with clinical trial methods.

Despite their potential benefits, there have been few published clinical trials of pain treatments that have used adaptive designs. It is possible that a number of analgesic trials in which futility was shown at interim analyses remain unpublished, and that some trials have used various types of adaptation but have not reported doing so in the published reports. Kalliomäki et al.^106^ have suggested that adaptive dose-finding designs can play an important role in early analgesic drug development. More generally, it can be anticipated that adaptive designs will also be used in studying whether sensory phenotyping or other biomarkers can predict which patients will show a greater response to analgesic treatment vs placebo.^38,80^

### 2.4. Selection of control and comparison treatments

In RCTs of analgesic medications, a matching inert placebo control—whether pills, saline injection, or topical vehicle cream or gel—very effectively controls for nonspecific influences on outcome, including placebo effects, regression to the mean, and spontaneous improvement. Identifying a control condition that does so in trials of invasive, psychosocial, and physical interventions is much more challenging. A variety of different approaches have been used, including sham surgery^137^ and sham devices that seem to be real but are missing a crucial therapeutic component (eg, an acupuncture needle that does not penetrate the skin or a stimulator that does not deliver stimulation).

For some treatments, the control condition can be “standard of care” or “treatment as usual” or even being placed on a waiting list for the active treatment. For both standard of care and waiting list control treatments, patients should be administered baseline and outcome assessments that are identical to and conducted at the same intervals as those administered to patients receiving the active treatment. Patients in both standard of care and waiting list control conditions can be expected to be receiving the treatment of their pain in the community, so when such designs are used, patients receiving the active investigational treatment should also be allowed to receive the same treatments (unless the objective of the trial is to compare the active treatment alone with what is typically used by patients in the community). For certain treatments and pain conditions, “add-on” trials can be used to show an additional benefit of a new treatment when added to an existing treatment. For example, patients who have had a partial response to a first-line medication can remain on that medication and be randomized to a new medication—presumably one with a different mechanism of action—or matching placebo to determine whether the new medication provides additional benefit.

It is often suggested that phase 2 RCTs examining analgesic and psychiatric medications with unknown or inconclusive efficacy should include a positive control with well-established efficacy in addition to a placebo control. Such a positive control makes it possible to demonstrate the assay sensitivity of the trial to detect the efficacy of the investigational treatment.^122^ That is, if neither the positive control nor the investigational treatment significantly differs from placebo, then the study can be considered a “failed trial” that lacked the assay sensitivity to demonstrate efficacy. However, if the positive control differs significantly from placebo and the investigational treatment does not, then it can be concluded that the trial had adequate assay sensitivity and that the investigational treatment lacks efficacy at the dosage studied and for the specific pain condition examined in the trial.

### 2.5. Allocation ratio

Most clinical trials of pain treatments allocate patients equally to the different treatment groups. It is also possible to randomize different percentages of patients to the different treatment groups, for example, randomizing twice as many patients to the active treatment as to placebo (2:1 allocation). Although there are various reasons to consider using such unequal allocation ratios, including enhancing the appeal of the trial to patients, sample sizes need to be increased for such trials to have equivalent statistical power as those with equal allocation ratios. The required increase in overall sample size for a trial with 2:1 vs 1:1 allocation, given a desired power of 80% or 90%, is modest, however (approximately 12%).

The results of meta-analyses of clinical trials of psychiatric and analgesic medications have suggested that when a greater percentage of patients are allocated to one or more active treatment groups than to placebo, there is typically greater improvement in the placebo groups and often smaller differences between the active treatments and placebo.^43,156^ This is thought to be due to placebo effects associated with patients having increased expectations of receiving active treatment. For example, patients participating in a placebo-controlled trial evaluating 3 different medication dosages would be aware that they have a 75% chance of being randomized to an active medication vs placebo. Of course, this assumes that the allocation ratio has been revealed to patients, either in the informed consent form, by the investigators, or on a clinical trial registration website (eg, www.clinicaltrials.gov). Such expectation effects, if present, might be prevented if patients are not aware of the allocation ratio because this information has not been revealed in consent forms, protocols, and websites. Blinding patients to the allocation ratio would not, however, be appropriate for RCTs that use unequal allocation to improve recruitment, in which case it would be important for patients to know that they have a greater probability of being randomized to active treatment than to placebo.

## 3. Endpoints and analyses

The FDA defines “treatment benefit” as the effect of a treatment on how “a patient survives, feels, or functions” and emphasizes that such benefit can be shown by an advantage either in efficacy or in safety, and that measures that do not directly capture effects on how patients feel, function, or survive are surrogate measures.^202^ This broad perspective is intended for the regulatory evaluation of drugs, biologics, and devices, and it is most applicable to later phase RCTs rather than phase 1 and 2 studies; nevertheless, it can also be used when considering other types of pain treatments, including such interventions as physical therapy, cognitive-behavioral therapy, and acupuncture. In designing an RCT of any of these interventions, a crucial decision involves selecting the specific outcomes that will be used to examine benefits on how patients feel, function, or survive. For trials of acute and chronic pain treatments, these outcomes will almost always include pain intensity and will often include physical and emotional function, as well as relevant aspects of safety.^39,191,194,198,200^ Several different types of outcomes have been used in clinical trials of pain treatments—quantitative measures (eg, ratings on a 0–10 numeric rating scale or a 10-cm visual analogue scale); counts of affected days or events (eg, number of days with migraine and number of trigeminal neuralgia paroxysms); categorical responses (eg, no, mild, moderate, or major improvement); and time to event outcomes (eg, number of days to mild or no pain).

### 3.1. Primary endpoints and analyses

The most common approach used in designing pain clinical trials is to select one efficacy outcome as the primary endpoint, for example, pain intensity as measured on a 0 to 10 numerical rating scale (NRS) at the end of the double-blind treatment period. However, there are multiple outcomes that can be evaluated in clinical trials of pain treatments, and depending on the specific trial objectives, it could be important to prespecify 2 or more primary endpoints.^200,205^ For example, an investigator might consider a novel treatment to be efficacious if improvement is shown on either a measure of pain intensity or a measure of physical function. Such “multiple primary endpoints” must be analyzed using an approach that controls the overall type I error probability, that is, the probability of rejecting the null hypothesis of no treatment effect for at least one of the endpoints when, in fact, the treatment has no effect. There are a variety of multiple comparison procedures that control the overall type I error probability at or below a specified level.^36,57^ Perhaps the most commonly used approach is the Bonferroni method, which uses a significance level of α/k for testing the null hypothesis of no treatment effect for each outcome variable, where α is the prespecified overall type I error probability and k is the number of outcome variables and, hence, the number of significance tests that will be conducted. Although the Bonferroni method can be easy to understand and implement, it is conservative and other procedures to address multiple testing have been developed that are generally more powerful.^36,57^

In some circumstances, an investigator might consider a novel treatment to be efficacious only if improvement is shown on 2 or more measures, for example, measures of both pain intensity and physical function. Such “coprimary” endpoints would all need to show a statistically significant difference in favor of the novel treatment for the treatment to be considered efficacious. When there are such coprimary endpoints, no adjustment is needed to control the type I error probability of falsely rejecting the null hypothesis of no treatment effect; this is because all the endpoints are required to show statistically significant differences in favor of the treatment for the treatment to be considered efficacious. However, the type II error probability (ie, the probability of failing to reject the null hypothesis when the treatment is actually beneficial) increases as the number of coprimary endpoints increases because every one of them is required to show a statistically significant difference in favor of the treatment. The sample size must therefore be increased to maintain adequate statistical power, with higher numbers of coprimary endpoints and lower correlations among them being associated with greater increases in the required sample size.^90,91,150,192^

For each outcome variable that is analyzed, prespecification of the precise definition of the outcome variable is necessary. Outcomes are typically measured at multiple time points, and the primary outcome variable can be defined at a single time point (eg, change from baseline to the end of double-blind follow-up) or across several time points. For example, the summed pain intensity difference is the sum (across time points) of the differences between baseline pain intensity and current pain intensity scores weighted by the time interval between ratings, and the total pain relief (TOTPAR) is the sum (across time points) of the relief scores weighted by the time interval between ratings; both measures are frequently used in clinical trials of treatments for acute pain.^51,183^ Of course, if outcomes at multiple time points are all deemed primary, then appropriate adjustment for multiple testing is needed. The form of the outcome variable also needs to be specified. For example, change from baseline in pain intensity measured using a 0 to 10 NRS can be specified as a continuous variable or as a dichotomous variable (eg, ≥30% pain reduction).

Other aspects of a trial design besides multiple primary endpoints can induce issues of multiplicity, and hence, approaches to deal with this are important to prespecify for the primary analysis. When there are more than 2 groups, for example, multiple dosages of a drug and placebo, the comparisons of primary interest need to be prespecified, with adjustment for multiple testing as appropriate. The inclusion of interim analyses also introduces issues of multiple testing, as described in section 6 below. Plans for examining treatment effects in subgroups (eg, based on quantitative sensory testing^33^) are usually reserved for secondary/exploratory analyses but should account for multiple testing if subgroup examination is planned as a primary objective.^80^

Decisions regarding endpoints and analyses should be made before beginning data analysis and prespecified in a statistical analysis plan and on a clinical trial registration website. The importance of prespecification of the statistical analysis plan cannot be overemphasized. Unfortunately, publications of RCTs of pain treatments are often missing crucial information about endpoints and analyses and whether they have been prespecified,^37,82,189^ which can make it difficult to adequately interpret the data given that selective reporting of outcomes or analyses or both may have occurred. Prespecification of the analyses should include the primary statistical model, the primary method for accommodating missing data (see section 5 below), and any adjustment for baseline covariates. It is standard practice to include variables used to stratify the randomization as covariates in the primary statistical model (eg, study center), and the baseline value of the outcome measure, if not used as a stratification variable, is also often included. Sometimes, analyses are performed that include baseline variables found to be distributed differently among the treatment groups as covariates in the statistical model; however, these and any other post hoc analyses should be considered secondary sensitivity analyses and clearly identified as such in all descriptions of the study results.

### 3.2. Secondary and exploratory endpoints and their analysis and interpretation

Secondary endpoints are included in RCTs to provide additional information about treatment benefit beyond that provided by the primary endpoint. Secondary endpoints can include outcomes that provide greater understanding of the overall treatment benefit—for example, if the prespecified primary outcome is a measure of pain intensity, then measures of physical function, mood, and sleep could be included in the trial to evaluate whether the treatment has beneficial effects on these aspects of health-related quality of life that are often adversely affected by pain. Other types of secondary endpoints include (1) separate components of a composite primary endpoint; (2) variables that can aid in understanding the mechanisms of action of the treatment; and (3) measures that relate to secondary hypotheses that are not major objectives of the trial.^32,200^

Analyses of secondary endpoints provide additional characterization and understanding of treatment effects but are not by themselves sufficient to confirm treatment efficacy in most circumstances. It is typical in clinical trials of pain treatments for the results of analyses of secondary endpoints and of other secondary analyses to be presented without any attention to the risk of type I error that results from multiple testing. The results of such analyses can provide a basis for subsequent research but cannot be considered a basis for concluding efficacy or for clinical decision making.

One common approach to analyzing primary and secondary endpoints and controlling the overall type I error probability involves sequential “gatekeeping” procedures that test a series of null hypotheses in a prespecified sequence.^200,205^ For example, a null hypothesis of no group difference in mean pain intensity could be tested first. If this null hypothesis cannot be rejected—that is, the group difference is not statistically significant—then testing stops. If the first null hypothesis is rejected, then a second null hypothesis of no treatment effect for, say, a physical functioning endpoint can be tested. If this second null hypothesis is rejected, then a third null hypothesis can be tested and so on. Testing stops at whatever point in the prespecified sequence a null hypothesis is encountered that cannot be rejected. Because of the hierarchical nature of the testing, hypotheses can be tested using the same significance level as that used in testing the previous hypothesis. Adjustment for multiple testing is not required when moving from one hypothesis to the next, and conclusions about each endpoint depend on whether the null hypotheses in the previous steps were rejected.^36^ Careful prespecification of the hierarchical sequence in which hypotheses are to be tested is critically important. It is possible, for example, for a treatment to have a profound effect on a secondary endpoint that is ranked low in the sequence, but the null hypothesis regarding the treatment effect on that endpoint would not even be tested if a null hypothesis concerning an endpoint that is ranked higher in the sequence is not rejected.

Exploratory endpoints are those endpoints included in an RCT that are typically not closely related to its primary objectives but that may provide worthwhile information about the treatment and provide the basis for the design of future studies. Endpoints that are prespecified as exploratory do not require any correction for multiplicity as long as no conclusions about efficacy will be drawn from the results of the analyses. When no approach to addressing multiple testing has been prespecified for the analysis of secondary endpoints—which is often true of clinical trials of pain treatments—the distinction between secondary and exploratory endpoints has no implications for data analysis but often reflects investigators' opinions about the importance of the endpoints.

Many RCTs collect a rich set of different types of data, and additional analyses might become of interest after the statistical analysis plan is final. Indeed, it has been noted that reviewers of articles can request that additional analyses be conducted and reported, and that such analyses can reflect knowledge of the data or interests of the reviewer rather than the original objectives of the clinical trial.^96^ Any unplanned analyses that were not prespecified should be clearly described as such in all clinical trial reports and publications, and the post hoc nature of these analyses must be considered when interpreting their results.^52^ Selective reporting of outcomes and analyses has been shown to lead to biased estimates of treatment benefit.^139,201^ Inappropriate data analysis and reporting can lead to erroneous conclusions, causing patients to receive ineffective treatments that could confer safety risks and unnecessary financial costs and personal burden.

### 3.3. Responder and composite endpoints

**Figure 1.**
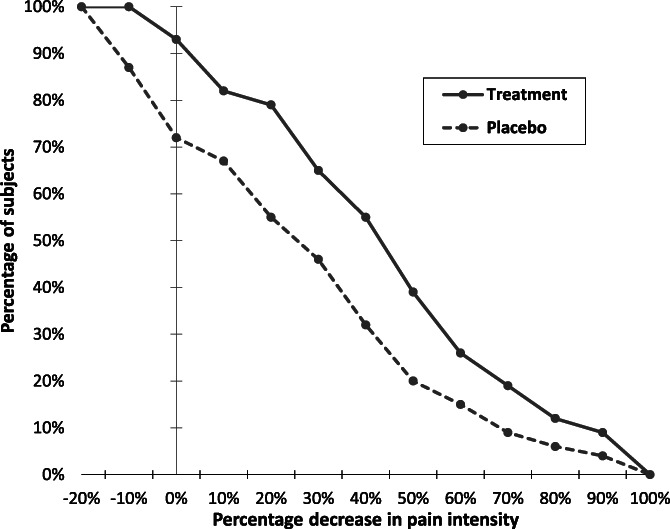
Empirical cumulative distribution functions for percentage changes from baseline ranging from small degrees of worsening through all possible degrees of improvement (reproduced from ref. 187).

There has been a great deal of attention devoted to the use of “responder” endpoints in RCTs of pain treatments. This is a result of multiple studies showing that reductions in pain of approximately 30% or greater and 50% or greater are, respectively, considered moderately and very clinically important by patients.^44,59^ These thresholds have been shown to apply to both acute and chronic pain and can be useful for evaluating whether a patient's pain reduction is meaningful, irrespective of whether the improvement reflects a true pharmacologic effect, a placebo effect, regression to the mean, or natural history. Reports of RCTs of pain treatments often present the percentage of patients in each of the treatment groups that have achieved one or both of these 2 “responder” definitions and test whether between-group differences in these percentages are statistically significant. In addition, because such categorizations are somewhat arbitrary, an increasing number of RCT reports also present the cumulative distribution functions for each treatment group, which makes it possible to examine group differences for every possible threshold of improvement (Fig. 1).^58^

Such “responder” definitions can also be used to calculate the number needed to treat (NNT), which is the inverse of the difference between the percentages of “responders” for the 2 treatment groups. For example, when 60% of patients administered a novel treatment and 40% of patients administered placebo have had ≥30% improvement in their pain, the NNT = 1/(0.6 − 0.4) = 5. The NNT can be thought of as the “number of patients who must be treated to generate one more success or one less failure than would have resulted had all persons been given the comparison treatment,”^119^ at least on average.

“Responder” endpoints and NNTs are widely used and can often facilitate meaningful interpretation of clinical trial results by clinicians and patients (section 7.2.1). However, they also have important limitations,^99^ including the substantial loss in statistical power that occurs when continuous endpoints are dichotomized.^60,116^ Larger sample sizes are needed for analyses of “responder” endpoints to have adequate power, as is also true for analyses of NNTs that are based on categorizing continuous data, as very often occurs in RCTs of pain treatments. In addition, NNTs can be misinterpreted with respect to their implications for clinical practice^171^ and are often thought to be the number of patients that a clinician would need to treat to get one positive response rather than the NNT to get one additional positive response beyond what would be obtained with the comparison treatment. As Senn^179,181^ has also emphasized, NNTs do not account for the heterogeneity of clinical trial participants: “Consider a trial comparing paracetamol with a placebo for treating tension headache. After 2 hours, 50% of people treated with the placebo are pain-free, as are 60% of those who were treated with paracetamol. The difference is 10%, and the NNT is 10. However, if paracetamol works for 100% of participants in 60% of the times they are treated, it will give the same NNT as if it works for 60% of the participants 100% of the time.”^181^

Composite endpoints can be used to combine multiple outcomes into a single measure and thereby test only a single hypothesis. Such endpoints have been used to address a variety of issues in RCTs^68^ and can be valuable when several endpoints are needed to adequately characterize the beneficial effects of treatment. A well-known example of a composite endpoint is the use of the ACR-20 in clinical trials of rheumatoid arthritis, in which patients are categorized as “responders” if there is a 20% improvement in tender/swollen joint counts and in 3 of 5 additional measures.^200^ Another type of composite endpoint would categorize patients as “responders” if, for example, they have either a prespecified level of improvement in pain (eg, ≥30% reduction) or a prespecified improvement in physical function.^157^ Ideally, the components of a composite endpoint should be associated with each other but not so highly associated that they provide nearly the same information that would be obtained from a single endpoint. Composite endpoints can also include both efficacy and safety outcomes for evaluation of the risk–benefit of a treatment, for example, by categorizing patients with respect to both clinical benefit and adverse events and then ranking the desirability of their joint outcomes.^56^ Such composites can incorporate associations among outcomes of interest and address competing risk challenges,^54^ for example, duration of acute pain being shorter in patients who die while recovering from their surgery.^27^

The major disadvantages of composite endpoints are that they generally do not permit conclusions about their specific components and can therefore be misinterpreted, that treatment effects may be limited to one or a few components that may be less meaningful, and that responses may even be qualitatively different for different components.^68^ For example, if a composite endpoint includes pain, physical functioning, and sleep, it is possible that a sedating treatment could have a meaningful benefit on pain and sleep but be associated with impaired functioning. Because composite endpoints can mask the beneficial or harmful effects of their individual components, it is generally recommended that separate analyses of each component be reported when results for composite endpoints are presented.^68,205^ If such analyses will be used to draw conclusions about the effects of treatment on individual components of the composite outcome, then prespecification of the approach that will be used for addressing multiple testing is necessary.

### 3.4. Adverse event assessment and analysis

Careful assessment of adverse events is an essential component of all clinical trials,^57,65^ including clinical trials of pain treatments.^191,199^ Adverse events can be assessed on the basis of spontaneous reports, by using a nonspecific approach in which patients are asked whether they have developed any new symptoms or health problems, or by a targeted approach in which patients are asked directly about specific symptoms (eg, dizziness and constipation), or a hybrid of the 2. The targeted approach is more sensitive for detecting specific symptoms. However, if relatively insignificant adverse events are more likely to be reported when patients are questioned about specific symptoms, the events ascertained on the basis of spontaneous reports or nonspecific questions may be more clinically relevant.

Regardless of which approach is used to collect adverse event and safety data, there are typically a large number of different events, laboratory values, and other measures that must be analyzed. There are a number of challenges in the analyses of safety data, including that events can be rare and can vary in the time when they first appear, that different patient characteristics can predict a greater risk of an event, and that multiple related or independent events can occur within individual patients. Most RCTs of pain treatments have sufficient power to detect a minimally important effect of treatment on the primary endpoint(s) but not necessarily to detect differences between the active and control treatments in adverse event rates and other safety outcomes. Because of this, efficacy trials can often have inadequate power to detect important treatment group differences in adverse event rates and other safety outcomes.^197^ Given the large number of events that are often examined, there is also an inflated probability of type I error as a result of conducting multiple significance tests of group differences. Depending on the specific circumstances, including the consequences of falsely concluding that there are no significant treatment-associated risks when there actually are, various procedures can be used to address the issue of multiple testing.^12^ Discussion of the complex issues involved in the appropriate analysis of adverse events and safety data is beyond the scope of this article, and other sources should be consulted.^57,65^

## 4. Sample size determination and statistical power

It is likely that one of the first issues that comes to mind when clinician investigators think about statistical aspects of clinical trials is the determination of the sample size. Unfortunately, published trials often do not adequately describe how this has been done, with one study finding that approximately two-thirds of RCTs in major medical journals fail to report all the information necessary for replication of the sample size determination or have inaccurate calculations or assumptions.^23^ Inadequate reporting of sample size calculations has also been found for clinical trials of pharmacologic and invasive pain treatments, with only two-thirds reporting a sample size calculation and only 38% of those trials describing all the information necessary to calculate the sample size.^135^

It has been argued that a larger sample size than is necessary is unethical because it exposes patients to safety risks and to potentially ineffective treatments or placebo.^124^ Too small a sample size can also be considered unethical given that patients are exposed to risks, but the result of the trial may be inconclusive.^3,89^ Small trials, however, may make a worthwhile contribution as early-phase studies with exploratory or feasibility objectives, for investigating rare diseases, and when there are commitments to include the results in meta-analyses.^46,89,132^

It is important to emphasize that the discussion of sample size determination in this section involves the decisions and calculations that are made before the trial begins enrollment, that are prespecified in the trial protocol, and that can be revised on the basis of prespecified interim analyses. After a trial is complete, analyses are sometimes conducted of what has been termed “observed” or “post hoc” statistical power. Such analyses, however, contribute no information beyond the reported *P* value and confidence interval for the treatment effect, and nonsignificant *P* values always correspond to relatively low observed power.^86,95,124^

### 4.1. Choosing the type I and type II error probabilities

The first step in calculating the sample size needed for a clinical trial is to choose the type I and type II error probabilities. For many phase 2 trials and most phase 3 trials, the type I error probability, or the probability of rejecting the null hypothesis when the treatment has no effect, is set at 5% (ie, a significance level of 0.05). This choice is arbitrary but has a long tradition in the medical, biological, and social sciences.^63^ Although setting the type I error probability at 5% is standard, in principle, the value should be set according to the consequences of making a type I error, that is, falsely declaring a treatment to be effective. There are circumstances where 10% or even higher can be used, such as in phase 2 studies in which an increased risk of a false-positive outcome can be offset by the knowledge that a statistically significant result will provide the basis for confirming efficacy in subsequent clinical trials rather than for a change in clinical practice.

A type II error is the failure to reject the null hypothesis when it should be rejected, that is, a failure to detect that a truly effective treatment is effective. With respect to clinical trials, a type II error will result in an efficacious treatment not showing efficacy and possibly being prematurely abandoned. Given this potential negative impact on public health and the very great costs, burdens, and risks associated with RCTs, the maximum type II error probability is usually 20%, with lower rates such as 10% preferred, especially in clinical trials involving a condition for which no treatment exists. The complement of type II error probability is statistical power, that is, the probability of rejecting the null hypothesis when it is false, and for most RCTs, it is prespecified within the range of 80% to 90%. Power is the probability of obtaining a statistically significant result when, for example, a truly efficacious treatment is compared with placebo. Power is greater with increases in sample size, type I error probability, and treatment effect magnitude and with decreases in the SD of the (continuous) outcome variable.

### 4.2. Treatment effect magnitude and variability

In addition to type I and type II error probability, sample size calculations for continuous outcome measures such as pain intensity depend on the magnitude of the treatment effect that trials are designed to detect and the variability of the outcomes. In many circumstances, identifying the treatment effect that the RCT should have adequate power to detect is the most challenging aspect of sample size determination. There are well-established cutoffs for the improvements in their pain that patients consider clinically meaningful,^44^ but determinations of clinically meaningful group differences depend on a variety of factors,^41^ not only the magnitude of the group difference but also whether other efficacious treatments are available for the specific condition, safety risks associated with the treatment, and other considerations discussed in more detail in section 7.

There have been systematic efforts to assess clinician opinions of clinically meaningful group differences for use in sample size determination,^85,193^ but such research has not been conducted for clinical trials of pain treatments. The results of a recent survey of clinical trialists^28^ and a systematic literature review^94^ found that diverse methods are used for specifying an important or realistic difference for sample size determination. These include opinion, pilot study results, anchor- and distribution-based methods, reviews and meta-analyses of previous trials, and cutoffs for small, medium, and large effect sizes. Not surprisingly, these different approaches are not always used appropriately, and guidance for specifying the “target difference” and reporting its justification has been published.^29,30^

Within the context of clinical trials of pain treatments, the primary endpoint is typically a continuous variable, pain intensity, based on a numerical or visual analogue scale; for example, the mean of daily pain ratings calculated over several days of treatment. One approach to sample size determination involves using a standardized effect size (SES), which for a parallel trial with 2 groups is the difference between the means of the groups divided by the SD, which is often assumed to be equal for the groups. For example, if patients administered an active treatment have a mean reduction in pain vs baseline of 3 points on a 0 to 10 scale, patients in the placebo group have a mean reduction of 2.0 points, and the SD of this outcome variable is 2.5, the SES is (3.0 − 2.0)/2.5 = 0.4.

A useful context for considering the magnitudes of such SESs and their effect on sample size requirements is provided by meta-analyses of RCTs of efficacious antidepressants for major depression, which have shown that the mean SES across trials submitted to the FDA and in the published literature is approximately 0.3.^83,110,111^ This is a modest treatment effect, one which would require an RCT to randomize 175 patients per group for 80% power with a 2-tailed significance level of 5%, not accounting for subject dropout. Comparable meta-analyses of analgesic RCTs do not exist; however, analyses of trials of efficacious chronic pain medications that examined measures of both average and worst pain intensity found mean SESs of approximately 0.3 for the most recent studies.^188,190^ Importantly, such estimates of treatment efficacy reflect not only the specific effect of the active treatment (eg, the pharmacologic activity of a medication) but also the assay sensitivity of the trial. Poor study design or execution can increase variability and compromise the ability of an RCT to detect a true treatment effect.

Given these considerations, how should the sample size for a clinical trial of a chronic pain treatment be determined? There are multiple possibilities, but all involve a decision regarding the minimum treatment effect that would be meaningful given a particular clinical context. As Kraemer et al.^119^ have emphasized, “It makes a difference whether the treatment is for a deadly disease like polio, or the common cold, and whether the treatment is risky and costly or perfectly safe and free.” It can be challenging for investigators to specify a minimum clinically meaningful treatment effect. For example, on a 0 to 10 pain intensity scale, is a group difference of 0.5 the minimum that would be clinically meaningful, or would a smaller difference be meaningful if the treatment is “perfectly safe”?^41^ In addition, investigators may not be able to specify the SD that should be used for the primary outcome measure given the specific pain condition and trial methods. In such circumstances, the results of previous RCTs of similar treatments could be used as a benchmark for identifying an SES that can be used in sample size determination; this assumes, of course, that the benefits shown in those trials can be considered meaningful given the specific treatment being examined and its context. It is important to emphasize, however, that information from a single, relatively small phase 2 trial or pilot study can be very misleading and should rarely be the primary basis for a sample size determination.^63,118^ The SESs from such trials—as compared with those from larger trials and meta-analyses—are less precisely estimated.

Whichever approach is used for determining the treatment effect used in a sample size calculation, it is advisable to be conservative regarding the assumptions on which it is based. For example, treatment effects in clinical trials of neuropathic pain appear to have declined over the past 3 to 4 decades,^62^ not because the treatments are less efficacious but presumably because the clinical trial methods and patient populations have changed. Hence, if treatment effects found in early studies are used as a basis for a sample size calculation, the trial might not have sufficient power to detect the effect that would be obtained by a trial conducted at present. Earlier clinical trials of analgesic medications were often conducted at a single expert site or by a relatively small number of sites within a given geographic area, whereas many current phase 3 analgesic trials have a large number of sites located in multiple countries. It would not be surprising if the SDs of outcome variables—which may depend, at least in part, on variability in patient training and investigator expertise—were greater at present than in the past. One implication of such temporal changes, especially for larger multinational trials, is that one should conservatively expect the treatment effect to be somewhat lower, and the variability as somewhat higher, than what would otherwise be anticipated. However, for trials that include a small number of highly select sites and experienced investigators, the temporal changes in treatment effect magnitudes might be less relevant. Given that there are multiple factors that must be considered in sample size determination that are often unknown or uncertain, performing an interim analysis to re-estimate the required sample size based on observed estimates of variability^70,168^ is a generally reasonable approach.

As noted above, the data and assumptions that formed the basis of the sample size determination (eg, SD of the outcome variable) are often inadequately described in publications of analgesic RCTs.^135^ Because such a lack of transparency can obstruct understanding of study methods, the sources and types of data and the assumptions made in sample size calculation should be routinely reported and justified given the circumstances of the trial.^29,30^

### 4.3. Additional considerations

The previous sections have used a standard parallel group clinical trial design with a normally distributed endpoint in discussing sample size determination. It is important to emphasize that other types of endpoints^31,163^ and different clinical trial designs will require additional considerations for sample size determination. For example, cross-over designs typically require fewer patients than parallel group trials because each patient is exposed to all treatments under study, and the reduction in sample size required to detect a given difference in outcome between treatment conditions can be substantial but will depend on the variability of within-patient differences.^178^ Noninferiority trials generally require larger sample sizes than superiority trials because the aim of the trial is to determine whether the difference in, say, the mean outcome between an established treatment and new treatment is no greater than a prespecified noninferiority margin, where this margin is typically chosen to be quite small.^22^ Sample size determination for trials with adaptive designs, including those with group sequential designs and other interim analyses, can be complex and needs to address potential inflation of the type I error probability due to multiple testing.^102^

For all clinical trials, missing data and treatment nonadherence will typically require that a larger sample size be randomized than would be needed for adequate power if data were complete for all patients and they adhered perfectly to the treatment regimen. Tests of hypotheses that patient subgroups—whether based on baseline demographic or clinical characteristics, or genotypic and phenotypic biomarkers—differ in their treatment effect will require a larger sample for adequate power.^80^ Such analyses of treatment-by-subgroup interactions will probably play an increasing role in RCTs of pain treatments given the substantial attention being devoted to the development of personalized (or “precision”) treatment approaches.^38,45,186^

In addition to the considerations for sample size determination of design complexity, missing data, nonadherence, and subgroup analyses, sample size requirements will be greater when multiple primary endpoints are prespecified. In RCTs with 2 treatment groups, unequal allocation ratios will also result in larger sample sizes because, for a given total sample size, power is greatest when group sizes are equal. The more complex the sample size determination, the more likely it is that conducting simulation studies to examine the effect of different trial design characteristics and assumptions on power would play a valuable role. Similarly, sensitivity analyses can be used to examine the trade-offs as different assumptions are varied.^57^ An example of such sensitivity analyses is examination of how the required sample size varies for different values of power (eg, 80% vs 90%), group difference(s) to be detected, and SD.

In addition to increasing the sample size, there are other approaches to increasing the power of an RCT to detect a given treatment effect. These include decreasing variability, for example, by increasing the reliability of the outcome measure^116,125^ or by using stratification or adjustment for covariates that are known to be associated with the outcome variable.^93,105,158^ As noted above, sample size requirements can be reduced by using cross-over designs where applicable.

## 5. Missing data and trial estimands

### 5.1. The problem of missing data

Subject dropout is common in clinical trials of pain treatments, particularly in those involving chronic pain conditions.^113^ This leads to missing data on outcome variables for subjects who do not complete follow-up. Data could be missing for reasons other than subject dropout as well, but this tends to be a relatively rare occurrence. Depending on the frequency of and reasons for the missing data, the interpretation of the trial results can be greatly affected. For this reason, it is important to deal with the problem of missing data both at the stage of study design (for purposes of prevention) and with the use of principled statistical methods.^121,146^

Historically, many clinical trials of pain interventions have used ad hoc methods to deal with missing data, including complete-case analysis (analyzing data from only trial completers), last observation carried forward, and baseline observation carried forward. These methods, although easy to implement, are known to have poor statistical properties.^141^ The broad use of these methods was not unique to the field of pain, leading the FDA to commission the National Research Council to produce a highly influential report that has led to improvements in the handling of missing data in practice.^146^ Important points emphasized in the report include the following: (1) taking steps at the stage of study design to prevent missing data is the best way to deal with the problem, particularly because statistical methods used to accommodate missing data all depend on untestable assumptions; (2) principled methods with good statistical properties have long existed and should be used to handle missing data; and (3) because of the dependence of statistical methods on untestable assumptions concerning the missing data, sensitivity analyses should be performed that examine how robust the results of the analyses concerning treatment effects are as those assumptions are varied.

### 5.2. Statistical methods to accommodate missing data

Principled methods based on sound theoretical development to accommodate missing data include direct likelihood methods (eg, the mixed model repeated measures [MMRM] approach^130^ and linear mixed effects models), multiple imputation,^20,173,176^ and inverse probability weighting (eg, weighted generalized estimating equations^140^). The MMRM approach appears to be the most widely used in pain clinical trials,^18^ particularly when group comparisons at a single time point are of primary interest. It uses maximum likelihood to estimate the model parameters (mean outcomes for each treatment group at each time point as well as variances and covariances among the outcomes at the different time points) taking into account the missing data.

All these approaches are known to be valid under the “missing at random” (MAR) assumption,^172^ that is, the probability of an observation being missing depends on only the observed data and not on the unobserved (missing) outcome that would have occurred. A useful illustrative example provided by Mehrotra et al.^138^ considers 2 trial participants in the same treatment group with similar baseline characteristics and an identical pattern of pain outcomes over time up until one of the participants drops out of the trial (the other completes follow-up). According to the MAR assumption, the participant who dropped out of the study would have had subsequent pain outcomes similar to those of the participant who completed the trial if she/he had remained in the trial. As noted above, however, this assumption cannot be tested.

Other methods for dealing with missing data have been proposed that treat all subjects who drop out as having had poor outcomes. In the context of “responder” analyses, those who fail to complete the trial are categorized as “nonresponders.”^142,143^ Another approach based on trimmed means was proposed by Permutt and Li.^160^ In this approach, subjects who fail to complete the trial are treated as having outcomes that are worse than those of anyone else in the trial cohort. After ordering the subjects in each treatment group from best to worst outcomes, a fixed percentage of subjects (eg, 30%) with the worst outcomes are omitted from each treatment group, where the percentage is chosen to be large enough so that all dropouts are omitted. The treatment effect is estimated as the group difference in mean outcome among the remaining sample, with resampling methods used to yield an inference concerning the treatment effect.^138,160^

Both of the above approaches assume that participants who drop out of the trial have poor outcomes. This assumption might be reasonable if subjects who drop out do so for reasons related to treatment (eg, intolerability) but might not be reasonable otherwise. For example, clinical trial participation often involves burdens for the subject, and it could be that a subject would be willing to remain on treatment in clinical practice but, for various reasons (eg, changes in life circumstances), is unwilling to continue to be followed regularly in a clinical trial. Assigning a poor outcome to this subject might not be warranted.^18^

### 5.3. Estimands

An important outgrowth of the National Research Council report was the considerable attention devoted to specification of the trial objectives, particularly the estimand of primary interest.^126,127,129,131,159,162^ The term “estimand” refers to the quantity that is of interest to estimate in the RCT based on the trial objectives and is the subject of the inference concerning the effect of treatment. The International Conference on Harmonization recently issued an addendum to the E9 guidance on Statistical Principles for Clinical Trials that outlines a framework for defining the estimand and emphasizes the role that the estimand plays in determining the design, conduct, analysis, and interpretation of the trial.^100^

An estimand is the population parameter to be estimated, which is defined in terms of the following components: (1) the treatment condition of interest and, if appropriate, the alternative treatment condition to which comparison will be made; (2) the target population; (3) the outcome variable; (4) handling of postrandomization (intercurrent) events; and (5) the population-level summary for the outcome variable.^100^ The most challenging aspect of this definition is the specification of how intercurrent events will be handled. In pain clinical trials, common examples of intercurrent events include discontinuation of the study intervention, reduction in dosage of the study intervention, use of permitted rescue treatment, use of a disallowed pain treatment, and change in the dosage of an allowed concomitant treatment.

The choice of the estimand influences many decisions regarding trial design, conduct, and analysis. For example, consider a randomized, double-blind, placebo-controlled clinical trial of a novel drug for painful diabetic peripheral neuropathy in which subjects will be followed for 12 weeks, with the primary outcome variable being the change from baseline to week 12 in pain intensity, as measured by the subject-rated average pain over 24 hours using a 0 to 10 NRS, itself averaged over the 7 days before each visit. The treatment conditions (novel treatment, placebo), outcome variable, and population-level summary of the outcome variable (group difference in mean 12-week change in pain intensity from baseline) are clearly specified. The eligibility criteria will define the target population of interest, including the level of pain intensity required at baseline, duration of the pain condition, outcome during a prerandomization run-in period (if applicable, in the case of an enrichment design; see section 2.3.3 above), and allowed concomitant medications for pain (if any).

Decisions then have to be made concerning how intercurrent events will be treated for purposes of defining the estimand. Consider 2 very different strategies in this regard:

*Strategy 1:* The group difference in the mean outcome for all randomized subjects that would have been obtained if all subjects tolerated and complied with treatment. In this case, any data collected after treatment discontinuation or dosage reduction, or use of a disallowed pain medication, would be omitted from the analysis, whereas data collected after use of permitted rescue medication or after a change in the dosage of an allowed concomitant treatment could be included in the analysis.

*Strategy 2:* The group difference in the mean outcome for all randomized subjects that would have been obtained if alternative treatments were not available other than those permitted by the protocol. In this case, in contrast to strategy 1, data collected after treatment discontinuation or dosage reduction would still be used in the analysis, as long as such data were actually collected and the subject did not initiate the use of a disallowed pain medication. As with strategy 1, data collected after use of permitted rescue medication or after a change in the dosage of an allowed concomitant treatment could be included in the analysis.

A major difference between the estimands defined in strategies 1 and 2 is the manner in which missing data should be handled. For strategy 1, it might make sense to assume that all the missing data are MAR if, in this case, a subject's trajectory after the intercurrent event could be assumed to be well modeled by the observed data, including their trajectory before the event. The analysis would be performed using a technique such as MMRM or a standard application of multiple imputation to deal with the missing data. For strategy 2, it might make sense to assume that the missing data are MAR for those for whom the data are missing for reasons that are unrelated to treatment. For other reasons for missing data, however, this would not be sensible. For someone who withdraws from trial participation due to, say, intolerability, one would not want to consider what that person's outcome would have been had the intercurrent event of intolerability not occurred. If the treatment has a beneficial effect on pain intensity but the subject cannot tolerate the treatment, the MAR assumption would continue to yield favorable pain outcomes for a subject in the novel treatment group, which would be inappropriately optimistic. Given the definition of the estimand, it might make more sense to impute the missing outcomes for such subjects as if they came from a similar placebo-treated subject who completed the trial. Also, it might be reasonable to assume that the missing data in the placebo group are MAR, regardless of the reason for the missing data.

It should be noted that what has historically been considered a pure “intention-to-treat” estimand would be the group difference in the mean outcome for all randomized subjects that would have been obtained regardless of any intercurrent events that may have occurred. Such an estimand would be associated with a pragmatic effectiveness scientific objective that might be of limited interest in some settings, such as placebo-controlled trials of novel interventions conducted for the purpose of regulatory approval, but may be of greater interest when estimating the effects in broader clinical practice populations. The collection of outcome data at week 12 from all subjects would be very important for this estimand given the difficulties in dealing with missing data in the absence of knowledge of what happened to subjects after dropout.

The above examples make it clear that the choice of the primary estimand depends on the scientific objective, the stage of treatment development, and the stakeholders.^127^ For a phase 3 trial, for example, a pharmaceutical company might prefer strategy 1 as more likely to yield a beneficial treatment effect in the trial, whereas a regulator might view strategy 2 as more clinically relevant. These examples also illustrate the importance of defining the scientific objective of the trial and the estimands of interest, as these have a major influence on elements of trial design, conduct, analysis, and interpretation. The emphasis on estimands represents a paradigm shift in the conduct of clinical trials. Historically, investigators would design the trial and specify a statistical analysis plan, including (often) simplistic methods to deal with missing data, and would be left with an estimate of treatment effect that was difficult to interpret. Having the study objective and estimand drive the design, conduct, and analysis of the trial leads to substantial clarity in its interpretation. The International Conference on Harmonization E9 Addendum^100^ provides excellent guidance and examples regarding choices of estimands; examples specific to clinical trials in pain are provided by Callegari et al.^19^ and Cai et al.^18^

### 5.4. Control-based imputation and sensitivity analyses

The example estimand in strategy 2 above requires that a principled method be used for dealing with missing data that is more flexible than those that assume MAR. A recently developed method of handling missing data that is coming into widespread use is control-based imputation.^21^ This method allows flexibility in the assumptions that one can make concerning the distribution of outcomes after dropout (or any intercurrent event that causes subsequent data to be treated as missing for purposes of analysis), given their observed outcomes, treatment group, and possibly other characteristics.

**Figure 2.**
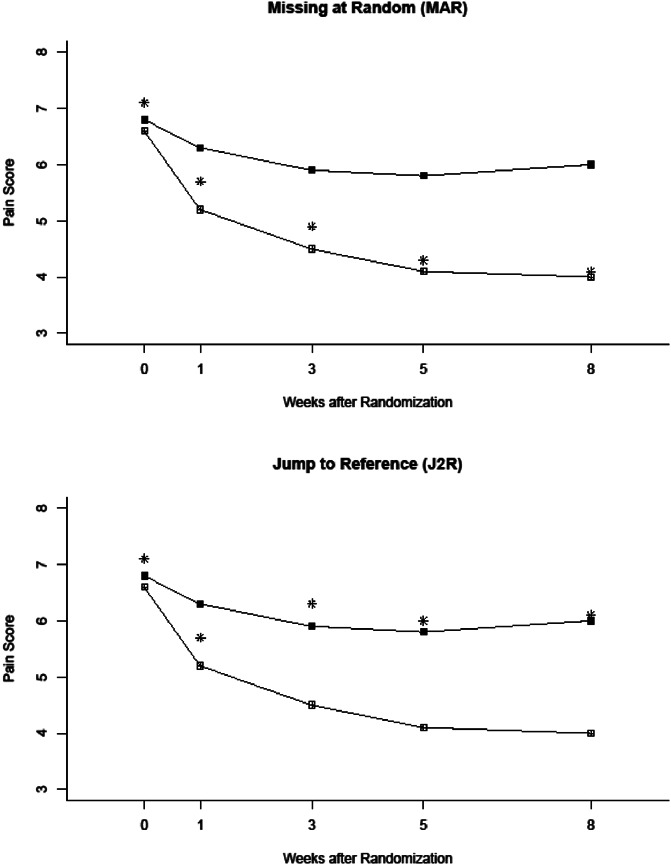
Illustration of typical imputed values for a subject in the treatment group that drops out of the trial after week 1 for different assumptions concerning the missingness mechanism: missing at random (MAR) and jump to reference (J2R). The black squares (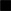) are the observed means in the placebo group, and the open squares (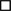) are the observed means in the treatment group. The asterisks represent either observed (weeks 0 and 1) or imputed (weeks 3, 5, and 8) values for the subject who dropped out of the trial.

To apply this approach to the estimand in strategy 2 above, one can assume that the missing data are MAR for subjects in the placebo group, an assumption commonly made in practice, and subjects in the novel treatment group who have missing data for reasons that are unrelated to treatment. For those with missing data for treatment-related reasons (eg, intolerability) in the novel treatment group, an assumption called “jump to reference” (J2R) can be used whereby outcomes after dropout (or an applicable intercurrent event) immediately switch to behave like those from similar subjects in the placebo group.^151^ Figure 2 illustrates typical imputations of missing pain intensity outcomes for a subject in the novel treatment group according to the MAR and J2R assumptions. Of course, these are just 2 examples of the many assumptions that can be made.^21,151^

Control-based imputation can be implemented with multiple imputation using pattern mixture models^21,151^ or a likelihood-based method that does not require explicit subject-level data imputation.^138^ Given its flexibility, the approach is particularly useful for performing sensitivity analyses to examine the robustness of the results of the primary analysis to assumptions concerning the missing data mechanism,^8,21,138,151^ but it might also be appropriate for the primary analysis of a clinical trial depending on the primary estimand of interest.^18,19^ The method should be used with caution when the clinical trial does not include a control group that is expected to be either comparable with or inferior to the active treatment groups under study with respect to outcomes.^151^

## 6. Data monitoring and interim analyses

### 6.1. Rationale for interim monitoring

In any clinical trial, it is important to use regular monitoring of study performance and data quality. Aspects to be monitored include adherence to the protocol on the part of both investigators and trial participants, enrollment progress, subject retention, and data quality.^74,108^ Periodic monitoring of individual clinical sites participating in the trial is also frequently performed, including adequacy of the study facilities, protocol adherence, documentation of informed consent, agreement between data entered on case report forms and data in source documents, accuracy and completeness of study records, appropriate regulatory reporting (eg, to the IRB and study sponsor), and storage and disposal of study drug, among other things. The purpose of regular monitoring is to identify problems in real time so that they can be quickly and effectively resolved and to prevent future similar problems from occurring.

The monitoring described above is often referred to as *blinded monitoring* because it is performed without regard to the treatment group to which the subjects have been assigned. This section will focus on *unblinded monitoring* of accumulating clinical trial data for the purpose of monitoring the safety of clinical trial participants and determining whether or not the accumulating evidence is sufficient to warrant modifying or halting the trial due to the efficacy, futility, or harm of the intervention(s) under investigation. There are ethical and economic reasons for such monitoring. If the data indicate that the risks to study participants are no longer acceptable, there is a clear ethical obligation to either modify or halt the trial. Similarly, if the data indicate that the intervention is efficacious, there may be an ethical obligation to inform all study participants, as well as the medical community and patients with the condition under study, of this finding. Finally, if the data indicate that the intervention is ineffective, then further exposure of study participants to the possible risks of continued participation may be unwarranted. Also, valuable resources can be saved by halting the trial on the basis of this information. Unblinded monitoring functions are typically performed by an independent committee, the role, composition, and function of which are described below.

### 6.2. Data and Safety Monitoring Boards

A Data and Safety Monitoring Board (DSMB) is a committee that is responsible for (1) safeguarding the interests of study participants, (2) preserving the integrity and credibility of the trial so that future patients may be optimally treated, and (3) ensuring that definitive and reliable trial results be made available in a timely manner to the medical community.^47^ It should be emphasized that the role of the DSMB is purely advisory to the trial sponsor; the ultimate responsibility for trial design, conduct, and reporting rests with the trial investigators and the sponsor.

A DSMB typically consists of members with a variety of expertise, including relevant medical, basic science, bioethical, and statistical expertise, and often includes others such as a patient advocate. Ideally, a knowledgeable and experienced chair would be chosen from among the members to guide the deliberations of the DSMB. A trial funded by a government sponsor (eg, US National Institutes of Health [NIH]) often includes a nonvoting sponsor representative on the DSMB. The importance of the independence of the DSMB members from the trial sponsor and investigators cannot be overstated given their role in being (ideally) the only individuals who have access to accumulating unblinded trial results. Members should be free of significant conflicts of interest, whether they are financial, intellectual, or personal, in order for the DSMB to be able to provide unbiased and objective advice to the sponsor and to preserve the integrity and credibility of the trial. It is essential that DSMB members maintain confidentiality of the unblinded information in their communications with the sponsor and study investigators.

The organization, role, and functioning of the DSMB are summarized in a formal charter. The charter describes the frequency and format of DSMB meetings, guidelines for interim analyses, extent of blinding of the DSMB, and rules for communication between the DSMB and the sponsor and study leadership, among other things. At the initial meeting of the DSMB, the draft charter and protocol, as well as the format of data reports to be prepared for subsequent meetings, are reviewed. Periodic meetings of the DSMB typically begin with an open session attended by the sponsor, representatives from the study team (eg, lead investigators and primary statistician), and DSMB members. In the open session, the DSMB is provided with an update on the study status (screening, recruitment, and retention), protocol changes (executed or proposed), and any problems that have occurred (eg, protocol violations) and how they have been resolved. The open session data report includes information on participant characteristics, participant disposition, information on data quality and site performance (recruitment and retention, data completeness and quality, timeliness of data entry, and query resolution), adverse events (including serious adverse events), and compliance with the intervention. This is followed by a closed session, attended by only DSMB members and a statistician appointed by the study team (DSMB liaison), during which a similar data report, with much of the above information broken down by the treatment group, is reviewed. The DSMB can request additional information from the statistician if desired. The DSMB then deliberates and formulates recommendations for the sponsor, after a formal vote among the members if necessary, and communicates these either by email or through a final open session with the sponsor and study team.

It is preferred that the statistician preparing closed session reports for the DSMB be someone who remains independent from the study team, that is, to not be the primary statistician for the trial, so that the primary statistician can remain blinded and provide unbiased advice to the study team as a member of the trial steering committee. It is equally important that the independent statistician be intimately familiar with the trial design and the data so that she/he can communicate effectively with the DSMB members during the closed sessions. The closed session DSMB reports often label the treatment groups with codes such as “A” and “B” without explicit reference to the identity of the treatment groups. In such cases, the DSMB would be given the ability to unblind themselves should this be necessary to make recommendations to the sponsor and study team.

The frequency of DSMB meetings is often linked to the rate of subject accrual, and quarterly or semiannual meetings are common choices. Because of the importance of rapid identification of potential safety issues associated with a treatment, some trials will require serious adverse events or other adverse events of special interest to be reported to the DSMB shortly after their occurrence (eg, within 24 hours of being reported) so that they can be monitored in real time.

The possible recommendations of the DSMB include (1) continue the trial without modification, (2) continue the trial after implementing a protocol modification, and (3) stop the trial. Possible reasons for a recommendation of early modification or termination of the trial include identification of an increased risk of harm in one of the treatment groups, clear evidence of efficacy for a treatment group, clear evidence of futility (lack of efficacy) for a treatment group, issues related to study performance (eg, slow accrual, poor data quality, and poor adherence to the protocol), relevant developments external to the trial, and loss of resources to complete the trial.

Not all clinical trials use or even require DSMBs. They tend to be used in trials of treatments for conditions that are associated with mortality or major morbidity or with progression of serious disease.^47^ Additional settings in which a DSMB might be appropriate include trials of high-risk treatments (including early-phase trials), trials involving vulnerable populations (eg, children and patients with dementia), and trials with a potentially large public health impact.^47^ Trials with complex adaptive elements such as sample size re-estimation, adding/dropping treatment arms, and seamless phase 2/3 designs would almost certainly benefit from the guidance of an independent DSMB. Both the NIH and the FDA have specific guidance on the need for, role, and function of an independent DSMB. Because publications of RCTs do not consistently report whether DSMBs were established and their membership, objectives, and processes,^78^ it is difficult to determine how often DSMBs have used for clinical trials of pain treatments and in what specific circumstances.

### 6.3. Interim analyses

Where applicable, it is important for a formal interim monitoring plan to be specified at trial initiation, including guidelines for modifying or stopping the trial based on evidence for the efficacy or futility of the treatment. Anticipated safety issues should be prespecified in the protocol. Formal guidelines for the monitoring of safety outcomes that would cover all the possible situations that might arise are more difficult to formulate given the higher degree of multiplicity involved. The clinical judgment of the DSMB is critical when considering treatment group imbalances in adverse events, particularly in the context of the severity of the events, the disease under study, and the potential benefits vs risks of the treatments being investigated.^53^ Guidance from the trial sponsor with respect to their philosophy on these issues can be valuable to the DSMB.^47^

Interim analyses for efficacy and futility, if desired, can be performed for the primary outcome variable with the use of a group sequential design.^102^ In addition to the planned primary analysis at the end of the trial, periodic analyses of the primary outcome variable would be performed as the trial is ongoing after a certain percentage of the information anticipated to be available at the scheduled end of the trial has been obtained. For a continuous outcome variable such as pain intensity, the percentage of information, called the *information fraction*, is simply the percentage of subjects scheduled to be enrolled in whom the primary outcome variable has been measured at the time of the interim analysis. For interim analyses for efficacy, the group sequential design adjusts the significance levels for each analysis to account for the number and timing of the analyses that will be performed to preserve the overall type I error probability at a prespecified level (eg, 5%). Another way to view this is that the design defines so-called stopping boundaries that dictate how large the value of the test statistic (eg, *t* statistic or Z statistic) needs to be to declare a statistically significant treatment group difference. Similarly, for interim analyses for futility, the group sequential design can incorporate well-defined stopping boundaries that preserve the overall type II error probability at a prespecified level (eg, 10% or 20%) under the effect size used to determine the sample size for the trial.

### 6.3.1. Group sequential boundaries

**Figure 3.**
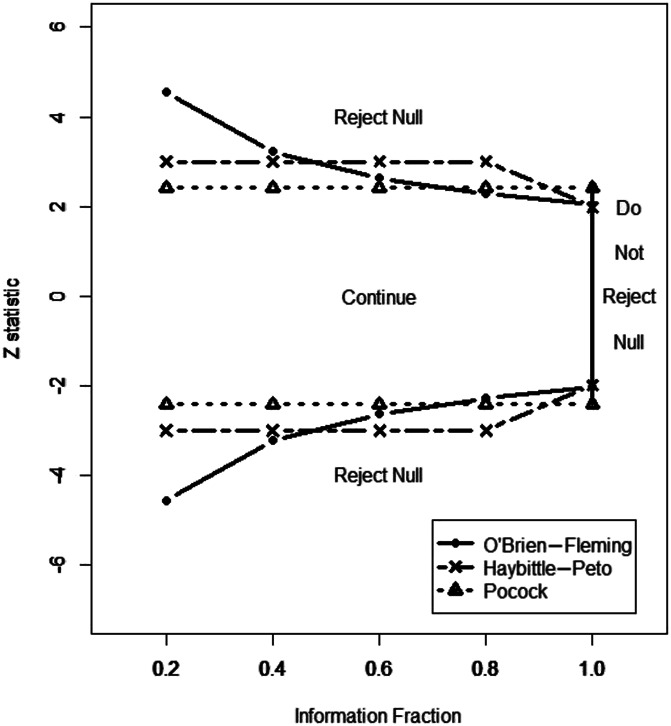
Illustration of 3 types of 2-sided efficacy boundaries for group sequential designs for a comparison of 2 groups. It is assumed that the test statistic (Z) is approximately normally distributed, and an overall 5% significance level is used. Interim analyses are to be performed after the primary outcome variable is available for 20%, 40%, 60%, 80%, and 100% of the enrolled subjects. At each interim analysis, if the Z statistic falls above the upper boundary or below the lower boundary, the null hypothesis of no treatment group difference is rejected; otherwise, the trial is continued.

Before the start of the trial, the boundaries to be used for interim monitoring need to be specified. This can be done by specifying so-called alpha- and beta-spending functions, the former for efficacy boundaries^120^ and the latter for futility boundaries.^155^ The number and timing of the interim analyses is typically prespecified as well, although these can be modified during the trial as long as the spending functions have been specified. Perhaps the most well-known stopping boundaries are the Haybittle–Peto boundary,^92,161^ the Pocock boundary,^164^ and the O'Brien–Fleming boundary.^149^ Examples of these boundaries (2-sided, 5% significance level) for efficacy are provided in Figure 3. In this example, the interim analyses for efficacy are scheduled to be performed after 20%, 40%, 60%, and 80% of the information on the primary outcome variable has been obtained. If the value of the test statistic (Z) falls above the upper boundary or below the lower boundary, the null hypothesis of no treatment group difference would be rejected.

*Haybittle-Peto boundary:* The boundary (|Z| = 3) is constant and very conservative for the 4 interim analyses, so very strong evidence against the null hypothesis would be required to stop the trial before its scheduled end. An advantage of this boundary is that the boundary at the final analysis (|Z| = 1.99) is very close to what it would be if no interim analyses were performed (|Z| = 1.96), that is, one pays a negligible price for having interim looks at the data if the trial continues to its scheduled end.

*Pocock boundary:* The boundary uses the same critical value (|Z| = 2.41) for all the analyses, including the final analysis. An advantage of this boundary is that one would be more likely to identify a treatment group difference at an interim analysis than if one were to use a highly conservative boundary such as Haybittle–Peto. On the other hand, if the trial continues to its scheduled end, the strength of the evidence required to reject the null hypothesis is greater, leading to an increased sample size requirement when planning the trial. This boundary might be best used if it is expected that a treatment group difference might be quite large and detectable early in the trial.

*O'Brien–Fleming boundary:* This boundary is highly conservative early in the trial when not much evidence has been accumulated and the results are not very stable, and becomes less conservative with time. This is intuitively appealing and is the principal reason for its widespread use in practice. A desirable property that it shares with the Haybittle–Peto boundary is that if the trial continues to its scheduled end, the boundary at the final analysis (|Z| = 2.04) is only slightly greater than what it would be if no interim analyses were performed. It is not as aggressive as the Pocock boundary in terms of detecting large treatment group differences early in the trial but becomes more aggressive than the Pocock boundary as the trial nears its scheduled end.

**Figure 4.**
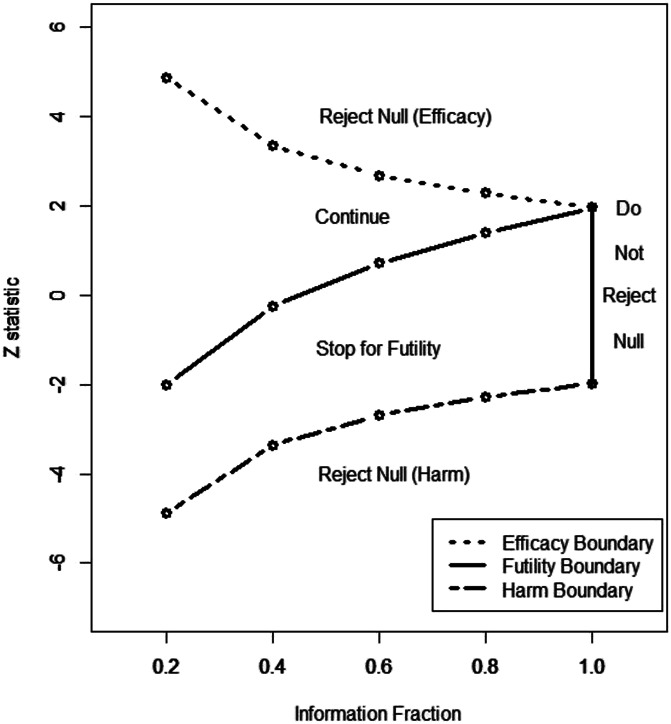
Illustration of a group sequential design with 2-sided O'Brien–Fleming efficacy boundaries and a 1-sided O'Brien–Fleming futility boundary for a comparison of an active treatment group and a placebo group. In this case, the lower efficacy boundary would indicate inferiority of active treatment (or harm) relative to placebo. It is assumed that the test statistic (Z) is approximately normally distributed, and an overall 5% significance level is used. Interim analyses are to be performed after the primary outcome variable is available for 20%, 40%, 60%, 80%, and 100% of the enrolled subjects. At each interim analysis, if the Z statistic falls above the upper boundary, the null hypothesis of no treatment group difference is rejected. If the Z statistic falls below the futility boundary, the trial is halted for futility (non-superiority) of the active treatment relative to placebo.

The above examples use 2-sided symmetric boundaries that are perhaps more applicable to clinical trials that are comparing 2 active treatments. For a trial comparing an active treatment with placebo, a one-sided stopping boundary for efficacy (using a significance level of, say, 2.5% instead of 5%) might be more appropriate because it would not typically be of interest to demonstrate that placebo was superior to active treatment. On the other hand, it is often of interest to stop the trial early if there is strong evidence that the treatment will not ultimately demonstrate a significant benefit in the final analysis, that is, to detect futility of the treatment. Figure 4 provides an example of an O'Brien–Fleming futility boundary superimposed on a 2-sided O'Brien–Fleming efficacy boundary, illustrating the fact that stopping for futility or lack of benefit of the treatment would occur sooner than stopping for demonstration of inferiority of treatment to placebo.

The above examples are limited in the sense that there is really no limit on the frequency and timing of the interim analyses (ie, they do not have to be equally spaced in terms of the information fraction), and there are rich families of alpha- and beta-spending functions from which to choose, not just the 3 illustrated above. Also, there are other tools that can be used for determining stopping boundaries such as conditional power/stochastic curtailment and Bayesian predictive power, but these can be formulated in terms of alpha- and beta-spending functions.^47,48^ There are additional approaches based on effect size, associated precision for estimating the effect, and prediction.^7,55,128^ Finally, it should be noted that estimators for treatment effects after early stopping are biased; however, methods exist to correct this bias.^49,112,212^

## 7. Interpretation of results

### 7.1. Statistical significance and confidence intervals

Once a trial has been completed and the data analyzed, careful interpretation of the results is necessary.^187^ To adequately evaluate the evidence of efficacy provided by a statistically significant primary analysis, several issues must be addressed. Foremost among these are whether the analysis that tested the primary hypothesis of the trial was prespecified and whether the possibility that multiple outcomes or analyses could have inflated the probability of a type I error and was addressed in a satisfactory manner. Additional important considerations involve whether there were any flaws or potentials for bias in the design and execution of the trial and whether the results for important secondary outcomes and subgroups were consistent with the results of the primary analysis.^167^ As discussed in the following sections, an evaluation of the extent to which the results of the clinical trial suggest that the treatment provides a clinically meaningful benefit is also very important.

**Figure 5.**
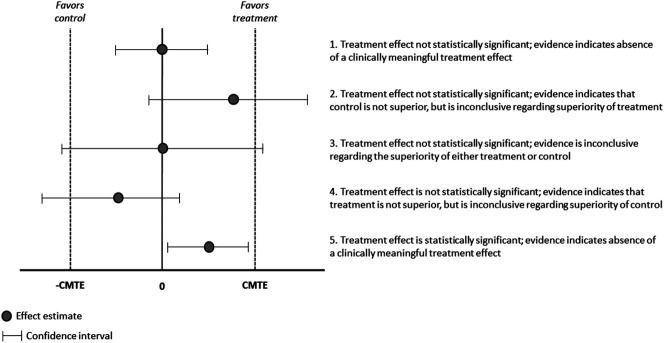
Interpretation of clinical trial results using confidence intervals for the treatment effect. CMTE, clinically meaningful treatment effect. Reproduced from reference 187 and adapted by permission from BMJ Publishing Group Limited: Gewandter JS, McDermott MP, Kitt RA, Chaudari J, Koch JG, Evans SR, Gross RA, Markman JD, Turk DC, Dworkin RH. Interpretation of CIs in clinical trials with non-significant results: systematic review and recommendations. BMJ Open 2017;7:e017288.

When the primary analysis of an RCT is not statistically significant, one possibility is that the treatment is truly efficacious but that the results of the trial failed to demonstrate that efficacy. This can happen for a variety of reasons, including a sample size that was too small to detect a meaningful treatment effect and problematic study design or execution that resulted in poor quality data and inadequate assay sensitivity.^43^ However, assuming that there was adequate study design and execution,^166^ it is important to evaluate whether the results are truly negative or whether they should be considered “inconclusive.” One important approach to making this determination involves examination of the confidence interval for the treatment effect. Specifically, if the confidence interval for the treatment effect does not contain what is considered a clinically meaningful treatment effect, then the trial can be considered negative, that is, the results failed to show clinically relevant efficacy (Fig. 5).^79,187^ However, if the confidence interval includes a clinically meaningful effect, then the trial results should be considered inconclusive and as providing the basis for further study to examine the treatment's hypothesized efficacy.^88^ Although biostatisticians have advocated this approach to interpreting nonsignificant results of clinical trials for many years, a recent review of RCTs published in general medical journals found that reporting and interpretation of confidence intervals was often problematic.^79^

Systematic reviews of RCTs in the general medical literature^15^ and for pharmacologic and invasive pain treatments^81^ have also shown that erroneous or misleading interpretations of statistically nonsignificant results are quite common. For example, it is often concluded that 2 interventions have comparable benefits when an RCT fails to show that one treatment is superior to another. This conclusion is only appropriate when a trial has been designed to test hypotheses of equivalence or noninferiority and not when the trial was designed to test the superiority of one treatment compared with another. Common examples of “spin” in the interpretation of RCTs with nonsignificant primary analyses include emphasizing statistically significant secondary analyses and focusing on improvements from baseline in the active treatment group rather than differences in such improvements between this group and the control or comparison group.

In recent years, there has been increasing recognition that *P* values are often misused and misinterpreted. For example, *P* values are frequently interpreted as the probability that a null hypothesis of no treatment effect is true. However, the *P* value is the probability that a treatment effect as large as or larger than that observed in the trial would occur if the null hypothesis were true. In addition, the interpretation that the results of an RCT constitute “proof” that the treatment is effective if they are statistically significant is flawed, as is the interpretation that the treatment is not effective if the results are not statistically significant (see the discussion above regarding “negative” vs “inconclusive” results). Importantly, the use of strict dichotomies with respect to *P* values to determine whether a treatment is efficacious can also be very problematic, with the level of evidence for treatment efficacy not being qualitatively different if, for example, *P* = 0.049 or if *P* = 0.051. Valuable discussions of important issues and challenges involving *P* values and their interpretation can be found in recent proposals and responses.^1,11,210,211^

### 7.2. What is a clinically important treatment benefit?

Statistically significant evidence of a treatment's efficacy in an RCT does not alone indicate that the magnitude of the treatment effect is clinically meaningful; for example, if the sample size is sufficiently large, very small group differences may be statistically significant, although they are clinically unimportant. The evaluation of clinical importance depends on whether the objective is to determine whether the improvements associated with treatment are important to individual patients or whether the group differences between treatments in an RCT are clinically important. It also depends on whether the benefits of a treatment are meaningful to society (eg, in reducing healthcare costs or increasing worker productivity). Such evaluations are obviously important and challenging but are beyond the scope of this article.

#### 7.2.1. Clinical importance of individual patient improvements

The results of numerous studies that have examined the magnitude of reductions in acute and chronic pain that are meaningful to patients have shown that decreases of ≥30% correspond to what patients generally consider “moderately important” improvements, whereas reductions of ≥50% can be considered “substantial” improvements.^44,59,152,153^ Although the percentage reduction in pain may be an informative approach to identifying thresholds for what patients consider clinically meaningful improvements, percentage change tends to be a highly variable measure that is not typically normally distributed and its use as an outcome measure is generally discouraged.^180,182,208^

Although meaningful reductions in pain intensity are, of course, important to patients with acute and chronic pain, patients typically consider other factors associated with a treatment in evaluating their overall improvement.^61,84,200^ For example, a marginal reduction in pain intensity could be accompanied by substantial improvements in sleep quality, mood, or physical function that, taken together, would be considered a major benefit by patients. Alternatively, apparently meaningful reductions in pain intensity could be accompanied by considerable side effects, with overall health-related quality of life unimproved or even worsened as a result. Unfortunately, such “trade-offs” between pain intensity and other factors associated with pain treatments have rarely been considered in evaluations of what levels of improvement patients consider meaningful.

#### 7.2.2. Clinical importance of group differences in a clinical trial

Evaluations of the magnitudes of pain reduction that individual patients consider clinically important are very often conflated with the determination of the magnitude of group differences between an active and a control treatment in an RCT that can be considered clinically important. The distinction between these 2 different concepts of clinical importance has been recognized for many years across a variety of therapeutic areas. It has been emphasized that the determination of the clinical importance of group differences in clinical trials should not be based on what improvements patients consider important for themselves but rather on a constellation of factors; these consist of the magnitude of the group difference in the trial, and also the broader context of the disease being treated, including whether other treatments are available, adverse events and safety risks associated with the treatment, and an overall evaluation of the benefit–risk profile, ideally as assessed by patients, clinicians, researchers, statisticians, and other stakeholders.^41,87,117,174^ For example, a decrease in pain intensity of ≥2 on a 0–10 NRS could be considered a clinically meaningful improvement for an individual patient, but this criterion should not necessarily be considered the difference between an active treatment and placebo for the effect of the treatment to be considered clinically important. The interpretation of meaningful change depends on whether it is being done “at a group level (where smaller changes may be interpreted as important) or at an individual level, where larger changes are required before they are confidently accepted as indicating a meaningful change.”^10^

Table 1 presents a number of factors that can be considered when evaluating the clinical importance of group differences in an RCT.^41^ The first consideration is that the differences must be statistically significant, which is generally a necessary criterion. In addition, the group difference in the primary outcome (eg, as assessed by the SES or another measure of treatment effect) can be compared with the treatment effects associated with other treatments that are considered to have clinically important benefits. Support for the clinical importance of the group difference in a clinical trial of a novel treatment is provided when the treatment effect is comparable to or greater than the effects seen with existing efficacious therapies. However, if the treatment effect found with the novel treatment is appreciably smaller than what has been found for existing therapies, it becomes essential to evaluate whether there are any other characteristics of the new treatment that might compensate for the modest treatment effect on the primary outcome. These characteristics include safety and tolerability, results for secondary efficacy outcomes including physical and emotional functioning, limitations of existing treatments, and the other factors listed in Table 1.

**Table 1:** Major factors to consider in determining the clinical importance of statistically significant group differences in clinical trials of pain treatments.

Treatment effect size for the primary outcome compared with available efficacious treatments
Safety and tolerability
Results for secondary efficacy endpoints (eg, improvements in physical or emotional functioning and health-related quality of life)
Rapidity of the onset of treatment benefit
Durability of treatment benefit
Limitations of available efficacious treatments
Different mechanism of action vs existing treatments
Cost, convenience, adherence, and other patient-centered factors
Other benefits (eg, few or no drug interactions and availability of predictive biomarker that is associated with an appreciable therapeutic benefit)

This table has been modified from ref. 41.

An evaluation of the overall clinical importance of the group differences found in a single RCT is one component of an overall assessment of the benefits vs risks of the treatment, which is usually determined on the basis of multiple studies and is, of course, a major consideration in regulatory decisions involving approval of medications and medical devices. There are a variety of approaches to conducting benefit–risk evaluations, ranging from the subjective but ideally evidence-based decisions made by advisory committees to quantitative multidimensional methods.^24,57,154^ Benefit–risk evaluations can be used to guide individual treatment decisions by patients and their clinicians and can also be valuable at the societal level as a basis for medical recommendations and policies, regulatory approvals, and reimbursement decisions. For benefit–risk evaluations to be meaningful, comprehensive analysis and presentation of the efficacy and safety data collected in RCTs is necessary. Unfortunately, as noted elsewhere in this article, there are inadequacies in the reporting of efficacy outcomes in clinical trials of pain treatments, and the descriptions and analyses of adverse events and safety risks in published pharmacologic^185,191^ and especially nonpharmacologic^98^ RCTs have also been problematic. Careful attention to CONSORT (www.consort-statement.org) and pain-specific guidelines^77,191^ for the reporting of clinical trial results can address these limitations of the existing literature.

## Disclosures

R.H. Dworkin has received in the past 5 years research grants and contracts from the FDA and the US NIH, and compensation for serving on advisory boards or consulting on clinical trial methods from Abide, Acadia, Adynxx, Analgesic Solutions, Aptinyx, Aquinox, Asahi Kasei, Astellas, AstraZeneca, Biogen, Biohaven, Boston Scientific, Braeburn, Celgene, Centrexion, Chromocell, Clexio, Concert, Coronado, Daiichi Sankyo, Decibel, Dong-A, Editas, Eli Lilly, Eupraxia, Glenmark, Grace, Hope, Hydra, Immune, Johnson & Johnson, Lotus Clinical Research, Mainstay, Medavante, Merck, Neumentum, Neurana, NeuroBo, Novaremed, Novartis, NSGene, Olatec, Periphagen, Pfizer, Phosphagenics, Quark, Reckitt Benckiser, Regenacy (also equity), Relmada, Sanifit, Scilex, Semnur, SK Life Sciences, Sollis, Spinifex, Syntrix, Teva, Thar, Theranexus, Trevena, Vertex, and Vizuri. S.R. Evans reports personal fees from Takeda/Millennium, personal fees from Pfizer, personal fees from Roche, personal fees from Novartis, personal fees from Achaogen, personal fees from the Huntington's Study Group, personal fees from ACTTION, personal fees from Genentech, personal fees from Amgen, personal fees from GSK, personal fees from the American Statistical Association, personal fees from the FDA, personal fees from Osaka University, personal fees from the National Cerebral and Cardiovascular Center of Japan, personal fees from the NIH, personal fees from the Society for Clinical Trials, personal fees from Statistical Communications in Infectious Diseases (DeGruyter), personal fees from AstraZeneca, personal fees from Teva, personal fees from the Austrian Breast & Colorectal Cancer Study Group (ABCSG)/Breast International Group (BIG) and the Alliance Foundation Trials (AFT), personal fees from Zeiss, personal fees from Dexcom, personal fees from the American Society for Microbiology, personal fees from Taylor and Francis, personal fees from Claret Medical, personal fees from Vir, personal fees from Arrevus, personal fees from Five Prime, personal fees from Shire, personal fees from Alexion, personal fees from Gilead, personal fees from Spark, personal fees from the Clinical Trials Transformation Initiative, personal fees from Nuvelution, personal fees from Tracon, personal fees from Deming Conference, personal fees from Antimicrobial Resistance and Stewardship Conference, personal fees from World Antimicrobial Congress, personal fees from WAVE, personal fees from Advantagene, personal fees from Braeburn, personal fees from Cardinal Health, personal fees from Lipocine, personal fees from Microbiotix, personal fees from Stryker, and grants from the NIAID/NIH, outside the submitted work; O. Mbowe has no conflicts to disclose; M.P. McDermott has been supported in the past 36 months by research grants from the NIH, FDA, NYSTEM, SMA Foundation, Cure SMA, Friedreich's Ataxia Research Alliance, Muscular Dystrophy Association, ALS Association, and PTC Therapeutics, has received compensation for consulting from Fulcrum Therapeutics, Inc and NeuroDerm, Ltd, and has served on Data and Safety Monitoring Boards (DSMBs) for NIH, AstraZeneca, Eli Lilly and Company, Catabasis Pharmaceuticals, Inc, Vaccinex, Inc, Cynapsus Therapeutics, Voyager Therapeutics, and Prilenia Therapeutics Development, Ltd.

Preparation of this article was supported by the Analgesic, Anesthetic, and Addiction Clinical Trial Translations, Innovations, Opportunities, and Networks (ACTTION) public–private partnership with the US FDA. ACTTION has received contracts, grants, or other revenue from the FDA, multiple pharmaceutical and device companies, philanthropy, and other sources. The views expressed in this article are those of the authors and no official endorsement by the FDA or the pharmaceutical and device companies that provided unrestricted grants to support the activities of the ACTTION public–private partnership should be inferred.
